# A universal ligand for lead coordination and tailored crystal growth in perovskite solar cells†

**DOI:** 10.1039/d3ee02344c

**Published:** 2024-01-09

**Authors:** Bowen Yang, Jiajia Suo, Dmitry Bogachuk, Waldemar Kaiser, Clemens Baretzky, Oussama Er-Raji, Georgios Loukeris, Asma A. Alothman, Edoardo Mosconi, Markus Kohlstädt, Uli Würfel, Filippo De Angelis, Anders Hagfeldt

**Affiliations:** a Department of Chemistry – Ångström Laboratory, Uppsala University SE-75120 Uppsala Sweden jiajia.suo@kemi.uu.se anders.hagfeldt@uu.se; b Laboratory of Photomolecular Science, Institute of Chemical Sciences and Engineering, School of Basic Sciences, Ecole Polytechnique Fédérale de Lausanne CH-1015 Lausanne Switzerland; c Fraunhofer Institute for Solar Energy Systems ISE, Heidenhofstr. 2 79110 Freiburg Germany; d Computational Laboratory for Hybrid/Organic Photovoltaics (CLHYO), Istituto CNR di Scienze e Tecnologie Chimiche “Giulio Natta” (CNR-SCITEC), Via Elce di Sotto 8 06123 Perugia Italy; e Freiburg Materials Research Center FMF, University of Freiburg, Stefan-Meier-Str. 21 79104 Freiburg Germany; f Department of Sustainable Systems Engineering (INATECH), Albert-Ludwigs-Universität Freiburg, Emmy-Noether-str. 2 79110 Freiburg Germany; g Chemistry Department, College of Science, King Saud University Riyadh 11451 Kingdom of Saudi Arabia; h Department of Chemistry, Biology and Biotechnology, University of Perugia, Via Elce di Sotto 8 06123 Perugia Italy; i SKKU Institute of Energy Science and Technology (SIEST), Sungkyunkwan University 440-746 Suwon Korea

## Abstract

Chemical environment and precursor-coordinating molecular interactions within a perovskite precursor solution can lead to important implications in structural defects and crystallization kinetics of a perovskite film. Thus, the opto-electronic quality of such films can be boosted by carefully fine-tuning the coordination chemistry of perovskite precursors *via* controllable introduction of additives, capable of forming intermediate complexes. In this work, we employed a new type of ligand, namely 1-phenylguanidine (PGua), which coordinates strongly with the PbI_2_ complexes in the perovskite precursor, forming new intermediate species. These strong interactions effectively retard the perovskite crystallization process and form homogeneous films with enlarged grain sizes and reduced density of defects. In combination with an interfacial treatment, the resulted champion devices exhibit a 24.6% efficiency with outstanding operational stability. Unprecedently, PGua can be applied in various PSCs with different perovskite compositions and even in both configurations: n–i–p and p–i–n, highlighting the universality of this ligand.

## Abstract 2

Phenylguanidine, a strong ligand in the precursor solution, retards crystallization to enlarge grain sizes and reduce defect density of a perovskite film, demonstrating excellent universality across various compositions.

## Graphical abstract

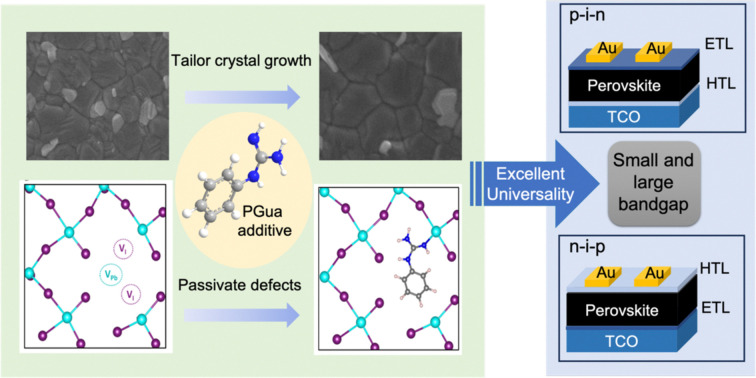

**Broader context**

Perovskite solar cells have shown remarkable progress with rapid increases in power conversion efficiency. However, the rapid crystal growth and the nature of solution processing induce various defects in the perovskite bulk and interface, which accelerate cell degradation. Additive engineering is an effective approach to address this issue. However, most reported additives show improvements in only one perovskite composition and device geometry. In this work, we propose a universal additive, namely 1-phenylguanidine (PGua), which coordinates lead iodide in the precursor solution to retard the perovskite crystallization process and form homogeneous films with enlarged grain sizes and reduced density of defects. As a result, the introduction of PGua unprecedently improves device performance for various perovskite compositions (small and large bandgaps) and configurations (n–i–p and p–i–n). Such a wide application provides vast opportunities for promoting PSC development and commercialization.

## Introduction

In recent years, the photovoltaic community witnessed the astonishingly rapid development of solar cells based on hybrid halide perovskites, which recently reached an impressive power conversion efficiency (PCE) of 26.0%.^1,2^ However, the rapid crystal growth as well as the nature of solution processing induce various impurities and defects at grain boundaries and interfaces in the produced perovskite films, which accelerate cell degradation and limit their commercialization.^3,4^ Since material engineering is a promising route to address this issue, various categories of additives (ionic liquids,^5,6^ Lewis acids^7^ and bases,^8–10^ ammonium salts,^2,11–15^ polymers,^16^*etc.*^3,4,17,18^) were developed to passivate the defects at the perovskite interfaces and grain boundaries. Among them, Lewis bases, with donated electrons, could efficiently coordinate with uncoordinated Pb atoms at interfaces and grain boundaries, enhancing the adhesion and improving the mechanical robustness of the perovskite film as well as the device stability.^4,10^ Unlike post-treatment of the perovskite surface, strong coordination interactions between a Lewis base and Pb^2+^ in precursor solution will significantly affect the crystallization behavior of the perovskite bulk, resulting in enlarged grain sizes and reduced trap density in the perovskite films, which, consequently, enhance the quality of the perovskite layer and improve the device properties.^19^

**Fig. 1:**
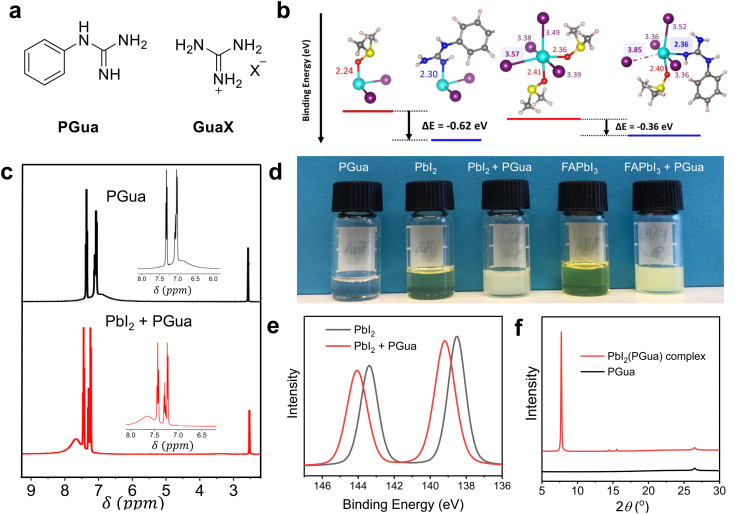
The interaction of PbI_2_ and PGua (a) chemical structure of PGua and GuaX; (b) binding energy of DMSO and PGua with PbI_2_ precursor complexes. Replacement of DMSO with PGua in a high-valent iodoplumbate [PbI_4_]^2−^ results in the release of iodide ions. The following color code is used for the atomic representations: purple, I; cyan, Pb; blue, N; red, O; yellow, S; gray, C; pinkish white, H. Distances between ligands and iodide and the Pb center are given in units of Å, highlighted in the color of the respective species. (c) ^1^H NMR spectra of PGua solution and the mixed solution of PGua and PbI_2_, which were dissolved in DMSO-*d*_6_; (d) photos of different DMSO solutions, the concentration in all cases is 0.1 M; (e) XPS core level signals of Pb 4f of PbI_2_ film and of PbI_2_(PGua) complex film; (f) XRD patterns of PGua and PbI_2_(PGua) complex films. The molar ratio of PGua and PbI_2_ is 1 : 1 in the above experiments (e) and (f).

Dimethyl sulfoxide (DMSO), as one of the most widely used solvents for perovskite precursor preparation, can coordinate with PbI_2_ as ligands to form PbI_2_(DMSO) intermediate species, retarding the crystallization of perovskite crystals and helping the formation of dense and uniform perovskite films.^3,4^ We propose that such a crystallization process can be further improved by introducing a small amount of a stronger Lewis base, for instance, guanidine derivatives. Since guanidine is a Y-shaped CN_3_ compound, within which the small, electron rich moiety core exhibits excellent coordination properties and shows monodentate σ-bond donating characteristics, it acts as a strong ligand to form guanidine–metal complexes with several metals, such as Co(ii), Cu(ii), Zn(ii), Pd(ii), Ni(ii) and Cr(iii).^20^ On the other hand, the multi amino nitrogen (N–H) moiety can bond strongly with halide anions, which could potentially provide additional benefits to regulate the perovskite crystallization kinetics.^3,4^ Hence, in this work, we designed and employed a new type of additive – guanidine derivative – namely 1-phenylguanidine (PGua), which consists of a guanidine group and a benzene ring, as shown in Fig. 1a. In contrast to the previously reported additives as well as commonly used guanidium ammonium salts (GuaX, X = I^−^, Br^−^, Cl^−^, SCN^−^, *etc.*), PGua shows a stronger interaction with uncoordinated lead and forms a stable intermediate complex with iodoplumbates in the precursor solution, which effectively retard the perovskite crystallization process, leading to the formation of a more homogeneous and stable perovskite film. This unique coordination chemistry investigated in this work by using PGua in the perovskite bulk results in a significant improvement of the opto-electronic properties of perovskite, which we have analyzed using a holistic photoluminescence-based characterization approach *via* a vast combination of spatially-, spectrally-, time-resolved and absolute photoluminescence-based measurements, which are coupled with our advanced recombination kinetics simulation. This deep analysis allowed us to extract multiple fundamental properties of perovskites and quantify the positive effect of PGua on the perovskite photo absorber itself, as well as on the perovskite solar cells. A champion device with a PCE of 24.6% and an outstanding long-term stability was obtained by the synergy of additive engineering with PGua and an interfacial passivation treatment with cyclohexylethylammonium iodide (CEAI). Remarkably, we highlight that PGua is a universal additive, which unprecedently improves device performance for various perovskite compositions (both small and large bandgaps) and configurations (both n–i–p and p–i–n).

## Results and discussion

### Perovskite solution chemistry

To gain insights into the role of PGua in the precursor solution, we analyze the interaction of PGua with [PbI_2_] complexes using density functional theory (DFT) calculations, mimicking the solution chemistry in the diluted precursor environments.^21,22^ PGua, representing a Lewis base, forms strong lead–nitrogen bonds between the nucleophilic NH group and the Pb^2+^ with binding energy of −1.49 eV, being 0.62 eV stronger than for DMSO, see Fig. 1b. Furthermore, we compare the binding energy of PGua with other neutral molecules that are frequently used as additives in precursor solutions,^23^ covering a wide range of additive chemistry *via* nitrogen (methylamine, PGua), oxygen (ethyl acetate, *N*-methyl-2-pyrrolidone, rhodamine), and sulfur (thiourea, methimazole) as Pb-coordinating moieties. We see that PGua is the strongest ligand among the considered additives with binding energy of *E*_rel_ = −1.49 eV, being at least 0.17 eV larger than for the remaining additives, see Table S1 (ESI†).

Considering solvated high-valent [PbI_4_(DMSO)_2_]^2−^ iodoplumbates, DFT calculations propose the facile exchange reaction of DMSO by PGua with a calculated spontaneous energetics (−0.36 eV), see Fig. 1b. Interestingly, introducing PGua into the high-valent iodoplumbate [PbI_4_]^2−^ results in the release of one I^−^ from the Pb^2+^ center, characterized by the Pb–I distance increase from 3.57 to 3.85 Å, highlighted in Fig. 1b. The suppression of high-valent iodoplumbates in diluted precursor solutions has been previously seen when adding lead chloride into precursor solutions.^22^ Notably, chloride ions, acting as strong ligands, are known to retard the crystallization of metal-halide perovskites, leading to more homogeneous perovskite layers with large grains,^24,25^ potentially through a sustained stoichiometry during cluster formation.^26^ As PGua represents the strongest coordinated ligand, we thus expect an increase in homogeneity, enhanced grain sizes and reduced defect densities when adding it in solution. Moreover, its phenyl group may further retard the crystallization due to increased steric hindrance.

Based on the DFT results, the additional PGua weakens the interaction between DMSO and PbI_2_ by intercalating into a closer position to the PbI_2_ framework. This strong interaction is further confirmed by nuclear magnetic resonance (^1^H NMR) measurements, using DMSO-*d*_6_ as the solvent. One broad peak is observed at around 6.8 ppm from the spectrum of the PGua solution, as shown in Fig. 1c, corresponding to the four active protons in the guanidine group. However, the chemical peak of the guanidine group shifts to a higher ppm (7.67 ppm) after mixing with PbI_2_ in solution (the molar ratio of PGua and PbI_2_ is 1 : 1), with accompanying downfield shifts of the proton peaks from the benzene ring. These obvious peak movements correspond to the variation of hydrogen nucleus electron cloud density of PGua, indicating that a strong coordination interaction occurs between PGua and PbI_2_. Similarly, the strong coordination interaction is observed from the mixed solution of FAPbI_3_ and PGua, as shown in Fig. S1a and b (ESI†). Moreover, as seen in Fig. 1d, a suspended solution can be clearly observed from the mixture of PGua and PbI_2_ with a concentration of 0.1 M. A similar phenomenon is observed when employing PGua in the FAPbI_3_ solution. Interestingly, the ultraviolet-visible (UV-vis) spectra (Fig. S1c, ESI†) show that after introducing PGua, the absorption peaks of both PbI_2_ and FAPbI_3_ solutions shift towards a similar lower wavelength of 300 nm, indicating the strong coordinating interaction between PGua and PbI_2_ while competing with DMSO in solution, which enables the formation of the same intermediate species in the two solutions regardless of the presence of FAI. This agrees with the above calculated formation energies of different complexes. We then carried out X-ray photoelectron spectroscopy (XPS), UV-vis absorbance spectroscopy and X-ray diffraction (XRD) measurements on the films fabricated from the above-mentioned solutions (Fig. 1d) to analyze the properties of the intermediate species and confirm its composition. Shifts in binding energies from the XPS results verifies the formation of the new intermediate complex. As indicated in Fig. 1e, the Pb 4f core level signals of the PbI_2_ film and the PGua–PbI_2_ mixture from XPS are found at 143.3 eV/138.4 eV and 144.0 eV/139.1 eV, respectively. Correspondingly, a slight shift towards higher binding energy of the N 1s signal of the PGua–PbI_2_ film is observed, compared to the pristine PGua film, as shown in Fig. S1d and e (ESI†). The transformation of PbI_2_ into PbI_2_(PGua) complex can also be evidenced by the UV-vis absorbance spectra (Fig. S1f, ESI†) and XRD measurements (Fig. 1f). In comparison with the PGua film, a new peak is observed at 7.7° from the film fabricated by the mixed solution (the molar ratio of PbI_2_ and PGua is 1 : 1). Considering the monodentate coordination nature of the guanidine compound,^20^ the absence of PbI_2_ peak (at 12.6°) indicates that the ratio between PbI_2_ and PGua in the newly formed complex is 1 : 1. The formation of the stable PbI_2_(PGua) intermediate complex is thus confirmed and is expected to reduce the uncoordinated lead atoms at interfaces and grain boundaries, retard the crystallization of perovskite, and thus improve the film quality and stability.

### Characterization of the perovskite films

In this work, we choose Cs_0.05_MA_0.1_FA_0.85_PbI_2.9_Br_0.1_·0.05PbI_2_ as the perovskite composition to fabricate high-quality and stable perovskite films,^13^ PGua-treated samples are fabricated with PGua (the concertation is 1.0 mg mL^−1^) doped perovskite precursor solution. Firstly, the existence of PGua in the as-prepared perovskite film can be realized from the appearance of the N 1s core level signals at 400.0 eV of the XPS measurements, as shown in Fig. S2a (ESI†), where both FA^+^ and MA^+^ from the reference film can be clearly distinguished (located at 400.4 eV and 401.8 eV, respectively, in Fig. S2a, ESI†). Accordingly, the Pb 4f core level signals of the PGua-treated film shift to a lower binding energy (Fig. S2b, ESI†) after the introduction of PGua. From the XRD measurements of the PGua-treated film (Fig. S3, ESI†), we note that an additional peak appears 7.7° consistent with the diffraction peak of the PbI_2_(PGua) complex shown in Fig. 1f. Moreover, the effect of PGua on modulating the perovskite crystallization growth can be evidenced by the scanning electron microscopy (SEM) images (top view in Fig. S4 (ESI†), cross-section in Fig. S5, ESI†), where larger and more uniform grains are observed from the PGua-treated films in comparison with the reference film, correlated with the improved coordination properties of the perovskite precursor. Remarkably, the strong coordination interaction between PGua and Pb^2+^ also enables a larger and improved surface coverage of the excess PbI_2_ crystals, that appear at the grain boundaries. As a result, it will help to reduce the trap density and further improve the film stability.^27,28^ Besides, we conducted UV-vis absorption spectra measurements of the perovskite films (with and without PGua) at various annealing times, as shown in Fig. S6 (ESI†). The results show that the formation of the black phase in PGua-treated perovskite films requires an extended annealing duration compared to the reference film. This observation suggests that the PbI_2_(PGua) complexes play a role in slowing down the perovskite crystallization process.

**Fig. 2:**
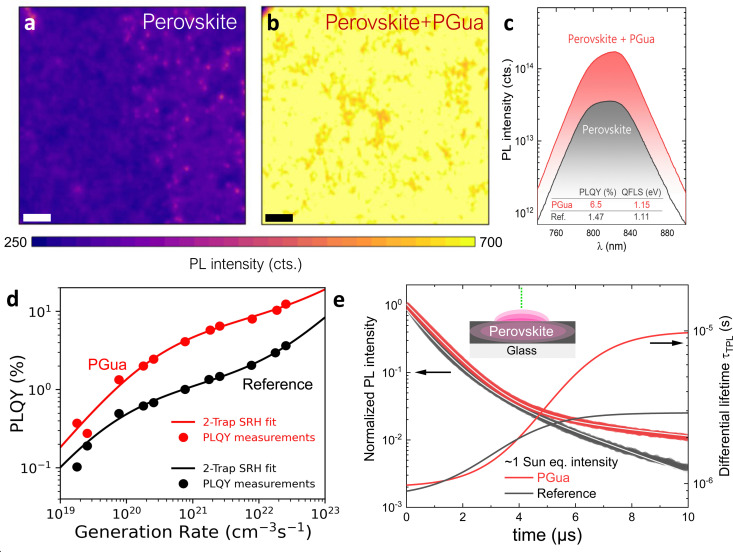
Opto-electronic properties of perovskite films. Spatially resolved photoluminescence (PL) measurements of (a) reference perovskite film and (b) perovskite films with PGua, scale bar – 6 μm. (c) Absolute PL measurement of a reference and PGua-treated perovskite films on glass with respective values for PLQY and QFLS. (d) Generation-dependent PLQY measurements fitted according to a two-trap level SRH model. (e) TRPL measurements of reference and PGua-treated perovskite films at ∼1 sun equivalent intensity and calculated differential lifetime based on the multi-exponential fits (white lines).

To evaluate the effects of PGua coordination during the perovskite crystallization on the opto-electronic properties of the film, we firstly performed steady-state spatially resolved photoluminescence (PL) measurements, revealing the spatial variation in radiative recombination of the reference and PGua-treated films. Fig. 2a shows that the reference film exhibits a significant spatial PL heterogeneity and an overall lower PL signal. In stark contrast, the PL map of the PGua-treated samples (Fig. 2b) shows a high and homogeneously distributed PL signal across the entire film, suggesting a lower rate of trap-induced non-radiative recombination and a more favorable crystallization kinetics. Furthermore, the photoluminescence quantum yield measurements (PLQY) in Fig. 2c show that at 1 sun-equivalent intensity, the PGua-treated sample (on glass) has a high PLQY of 6.5%, whereas it is only 1.47% in the reference films. Consequently, the quasi-Fermi level splitting (QFLS) extracted from the PLQY measurements *via* Ross relation^29^ was found to be 1.15 eV and 1.11 eV for the PGua and reference films, respectively, highlighting effective suppression of non-radiative recombination in perovskite films by the introduction of PGua.

To quantify the recombination rates and the effect of PGua on the opto-electronic properties of the perovskite films, we also performed light intensity-dependent PLQY measurements (Fig. S7, ESI†) and compared them with the numerical solutions for the generation–recombination rate equations under steady-state conditions. In this case, we used a two-trap level Shockley–Read–Hall model. Fig. 2d demonstrates that the PLQY of the PGua-treated samples is consistently higher than that of the reference samples under all generation rates. From the fits of the measured PLQY to the SRH model, the suppression of non-radiative recombination can be explained by a shift of the shallow trap level closer to the closest band edge, thus reducing recombination through the shallow trap level when PGua is introduced into the perovskite solution. Description of the implemented two-trap level SRH model and fitted parameters can be found in the Note S1 (ESI†).

The time-resolved PL measurements in Fig. 2e show that after the generation stops, the PL of treated films decays much slower than that of reference films, highlighting the strongly reduced non-radiative recombination rate in the former case, which agrees with the PLQY measurements. Furthermore, the differential lifetime *τ*_TPL_ calculated from the decay fits (details are shown in Note S2, ESI†) illustrates that, the rate of non-radiative recombination is significantly reduced in the PGua-treated films leading to SRH lifetimes approaching 10 μs. The light intensity-dependent TRPL measurements (Fig. S9, ESI†) confirm that the non-radiative recombination is higher in reference perovskite films under all the generation rates. Notably, a shift from the mono- to multi-exponential decay type happens at much lower intensities (0.001–0.01 sun) in the PGua samples than in the reference ones. This early transition to the higher-order radiative and SRH recombination mechanisms highlights that they start to dominate under these injection conditions. In contrast, the mono-exponential decay of the reference films at nearly all the intensities indicates that the trap-induced SRH recombination processes are dominant. Overall, it is remarkable to see that even moderate quantities of PGua in the perovskite solution can effectively suppress non-radiative recombination in the perovskite films and prolong charge carrier lifetime, which is essential for manufacturing high-performing PSCs.

**Fig. 3:**
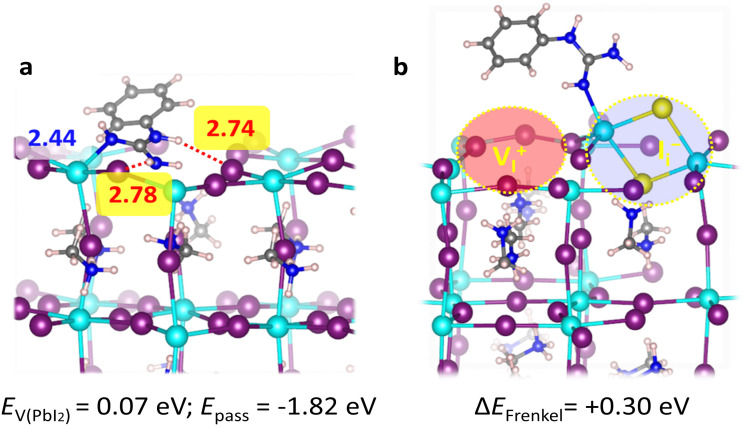
DFT calculations on the passivation mechanism. Optimized geometry structures of the perovskite slabs passivate with one PGua molecule for (a) the surface with one PbI_2_ vacancies (V_PbI_2__), see Fig. S10 (ESI†) for top view, and (b) for the surface with iodine Frenkel defects (V^+^_I_/I^−^_i_). Main interacting atomic distances and location of the defects are also highlighted with dashed circles. Following color code is used for the atomic representations: purple, I; cyan, Pb; blue, N; red, O; yellow, S; gray, C; pinkish white, H.

Except for retarding the perovskite film crystallization process with enlarged grain sizes, the exact mechanisms of trap state reduction induced by the presence of PGua could be understood in-depth *via* our DFT calculations of the frequently occurring defects in the perovskite films. First, we consider two frequent surface defects with low formation energies that may appear in lead-halide perovskites, PbI_2_ vacancies (V_PbI_2__)^30,31^ and iodine Frenkel defects (V^+^_I_/I^−^_i_),^32,33^ see Fig. 3 and Fig. S10 (ESI†). Both defects show low formation energies of 0.07 eV (V_PbI_2__) and 0.09 eV (V^+^_I_/I^−^_i_) at grain surfaces due to the presence of undercoordinated surface Pb ions. Note that we expect that both defects are present in perovskite grain boundaries due to incomplete crystallization, as recently shown for the iodine Frenkel defect.^34^ PGua shows excellent passivation abilities of the V_PbI2_, with passivation energy of −1.82 eV, by strong binding with the undercoordinated surface Pb ions and additional hydrogen-bonding interaction with the undercoordinated surface iodide ions. Moreover, we observe a substantial destabilization of iodine Frenkel defect formation by 0.3 eV, which can inhibit iodine defect formation and consequently suppress non-radiative recombination. Thus, our analysis further suggests a potential integration of small amounts of PGua in the perovskite bulk without introducing trap states in the band gap (see Fig. S11, ESI†), which potentially can suppress bulk defects. Comparing various additives, PGua shows ideal passivation abilities at grain surfaces (Table S2, ESI†) and bulk (Table S3, ESI†) due to its strong Lewis base nature.

### Photovoltaic performance, stability and universality of the PSCs

**Fig. 4:**
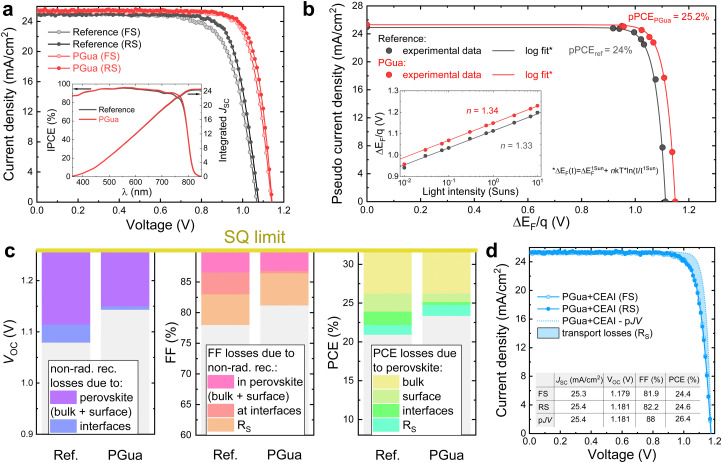
PSCs photovoltaic performance. (a) *JV*-curves of reference and PGua treated perovskite films obtained from forward voltage sweep (FS) and reverse voltage sweep (RS). The inset shows the incident photon to electron conversion efficiency (IPCE) with an integrated *J*_SC_. (b) Pseudo *JV*-curves of reference and PGua treated perovskite films on glass reconstructed from the intensity-dependent QFLS measurements (inset), showing potential PCE of the corresponding films. (c) *V*_oc_, FF and PCE loss analysis based on the data extracted from the light intensity-dependent measurements (complementary data can be found in Note S4, ESI†). (d) *JV*- and pseudo *JV*-curves and characteristics of the manufactured PSC with PGua and interfacial passivation with CEAI.

To investigate the effect of PGua on the device performance, PSCs based on FTO glass/cp-TiO_2_/mp-TiO_2_/Cs_0.05_MA_0.1_FA_0.85_PbI_2.9_Br_0.1_·0.05PbI_2_/spiro-MeOTAD/Au were fabricated with different concentrations of PGua in the precursor solution. Compared with the reference devices, the enhancement in device performance upon PGua treatment can be attributed to the increase of all photovoltaic parameters, especially the open-circuit voltage (*V*_OC_) and fill factor (FF). As shown in Fig. 4a and Fig. S13 (ESI†), cells fabricated with 1 mg mL^−1^ PGua show the best results, with a champion PCE of 23.41%, *J*_SC_ of 25.29 mA cm^−2^, *V*_OC_ of 1.142 V, and FF of 81.1% (Fig. 4a and Table S4, ESI†). The slight increase in *J*_SC_ by 0.3 mA cm^−2^ obtained from the *JV*-measurements is consistent with a slightly higher absorption in the long wavelength region, as seen from the inset of Fig. 4a. We attribute this to the better light-absorbing properties of the PGua-treated perovskite films, based on the measured absorption coefficient and Tauc plot in Fig. S14 (ESI†). To investigate the origin of high FF and *V*_OC_ in PGua-treated films, we measured the light intensity-dependent *V*_OC_ of treated and reference cells (Fig. S15a, ESI†) to construct their respective pseudo *JV*-curves, from which we quantified the FF losses stemming from the series resistance (Fig. S15b, ESI†). From the difference between the pseudo FF and the FF at the radiative limit, we found the non-radiative FF loss in reference cells to be 7.24%, whereas in PGua-samples it is only 3.8%. To disentangle the contributions of the charge-selective layers to the non-radiative FF losses, we used the generation-dependent PLQY (and QFLS) data (Fig. 4b inset) to construct a pseudo-*JV* curve of treated and non-treated perovskite films (Fig. 4b). Such measurement allows the quantification of the potential QFLS of a semiconductor in the absence of any additional non-radiative recombination losses due to interfaces (which are normally present in the complete solar cells).^35^ By knowing the QFLS at different light intensities and ideality factor (*n*), pseudo *JV*-curves of neat perovskite films could be fitted to the measured values to produce a *JV*-characteristic of a solar cell in the absence of interfacial non-radiative losses, energy band misalignment, series resistance losses and optical losses caused by the charge-selective layers. Despite the ideality factor being the same with or without PGua, the PGua-treated samples could in principle achieve a PCE of 25.2%.

Remarkably, the measured *V*_OC_ of the PGua-cell is very close to the QFLS/*q* extracted from the PLQY spectrum of the PGua-films at 1 sun-equivalent intensity of 1.149 V. This clearly demonstrates that the presence of PGua significantly reduces the non-radiative recombination losses at the interfaces between perovskite and charge-selective layers. In contrast, 35 mV are lost due to the defect-induced recombination at the interfaces in reference cells (Fig. 4c). As can be seen from the FF loss graph in Fig. 4c, the series resistance (*R*_s_) remains nearly unchanged in the cells with or without PGua. However, the interfacial non-radiative recombination FF loss is strongly suppressed by PGua passivation resulting in the total improvement in FF by 3.2%, which is the main reason for the observed FF improvement in the cells with PGua.

Furthermore, we manufactured additional PSCs with PGua-treated perovskite and same cell structure but with an additional interfacial layer of a cyclohexylethylammonium iodide (CEAI), which was investigated in earlier work.^36^ The measured *JV* and pseudo-*JV* curves of the champion cell (Fig. 4d) demonstrate an impressive PCE of 24.6%, with a *V*_oc_ of 1.181 V, which is <80 mV away from the SQ limit of 1.259 V at this bandgap (more details can be found in Note S4, ESI†). In addition, this cell has a remarkably high FF of 82.2%, which is consistent with our earlier finding that CEAI suppresses the non-radiative recombination FF losses. Based on the pseudo PCE of 26.4% in the case of CEAI and PGua-treated cells, we attribute the remaining PCE losses of ∼5.5% (relatively to the SQ limit) to non-radiative recombination losses in the perovskite bulk, since PGua and CEAI passivate the perovskite surface states, and the only remaining loss besides the series resistance is the non-radiative recombination in the bulk. This assumption allows us to show in Fig. 4c that PGua reduces the losses due to surface defect-induced recombination in neat perovskite films corresponding to a potential 1.25% absolute efficiency gain.

We note that achieving a high product of *V*_OC_ and FF is currently a major challenge in PSCs, since very effective passivation strategies often hinder charge transport (*e.g.* trioctylphosphine oxide) and therefore can result in high *V*_OC_ but low FF. Conversely, non-passivated perovskite might have a fast carrier transfer to the charge transport layers but suffer from significant non-radiative losses. Here, we demonstrate that PGua samples (together with CEAI passivation) result in a high product of *V*_OC_ × FF, which is nearly 85.5% of the SQ limit for semiconductor with such bandgap. Fig. S16 (ESI†) demonstrates that our champion device displays superior product of *V*_OC_ and FF, when compared with the perovskite database collected from literature reports,^37^ highlighting its excellent opto-electronic properties.

**Fig. 5:**
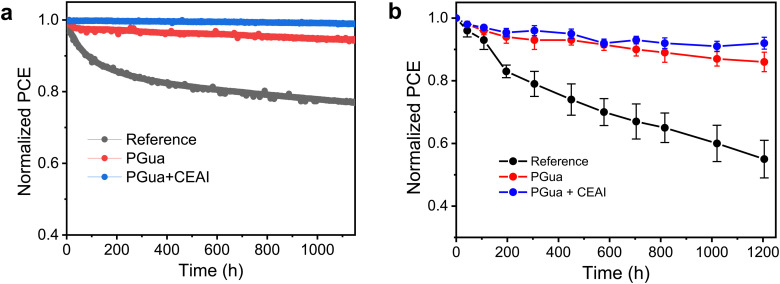
The stability test of PSCs. (a) Long-term stability of unencapsulated devices from different conditions under MPPT in N_2_ atmosphere at room temperature; and (b) thermal stability of unencapsulated devices from different conditions at 85 °C in the N_2_ atmosphere, five devices for each condition.

Furthermore, the long-term stability of devices with and without PGua treatment were monitored under different conditions. As shown in Fig. 5a, the reference device shows more than 20% relative PCE loss after 1150 h maximum power point tracking ageing under continuous 1-sun illumination, while the PGua treated devices retain 95% of their initial value. In addition, interfacial treatment *via* introduction of CEAI to remove the PbI_2_ at the perovskite surface could further improve the device stability.^38,39^ The combined passivated device shows impressively less than 1% loss during the same ageing condition, yielding an excellent theoretical *T*_80_ of over 3 years, as shown in Fig. S17 (ESI†). Moreover, the devices were subjected to under 85 °C temperature conditions to evaluate the thermal stability by replacing the thermal stable hole transporter layer with poly[bis(4-phenyl)(2,4,6-trimethylphenyl)amine] (PTAA). The introduction of PGua could also help to improve the device thermal stability, especially the CEAI combined devices retained 92% of its initial PCE, whereas the control devices show almost 50% loss after more than 1200 h, as shown in Fig. 5b.

Finally, we fabricated PSCs with different perovskite compositions to investigate the effect of PGua additives when changing the A-site cations and the ratio of halide ions. Encouragingly, Table 1 demonstrates that PGua can be applied to various kinds of PSCs with different perovskite compositions and even in both configurations: n–i–p and p–i–n, which is barely the case for additives reported in the literature, highlighting the impressive universality of this remarkable additive. The *JV*-curves of the champion devices and statistics of photovoltaic parameters can be found in Fig. S18–S24 (ESI†). We attribute the excellent universality of PGua in various lead-containing perovskite systems to its strong interaction with lead compounds in the perovskite precursor solution, which effectively retards the perovskite crystallization process, resulting in the formation of a more uniform film with increased perovskite grain sizes and reduced defects.

**Table 1:** Photovoltaic parameters of champion PSCs with and without PGua

Perovskite composition	Architecture	Additive	*J*_SC_ (mA cm^−2^)	*V*_OC_ (V)	FF (%)	PCE (%)
MA_0.1_FA_0.85_Cs_0.05_PbI_2.9_Br_0.1_·0.05PbI_2_	n–i–p	w/o PGua	24.97	1.08	77.9	21.0
		w/ PGua	25.29	1.14	81.1	23.4
MAPbI_3_·0.05PbI_2_	n–i–p	w/o PGua	24.24	1.08	76.2	20.0
		w/ PGua	24.43	1.10	78.5	21.1
MAPbBr_3_·0.05PbBr_2_	n–i–p	w/o PGua	7.86	1.42	58.7	6.6
		w/ PGua	8.12	1.52	68.3	8.4
MA_0.1_FA_0.85_Cs_0.05_PbI_2.9_Br_0.1_·0.05PbI_2_	p–i–n	w/o PGua	21.60	1.06	81.6	18.7
		w/ PGua	23.20	1.08	81.3	20.4
FA_0.83_Cs_0.17_PbI_1.8_Br_1.2_	p–i–n	w/o PGua	11.00	1.14	76.1	9.5
		w/ PGua	16.30	1.22	67.8	13.4

## Conclusions

Taking experimental analysis together with DFT calculations, we prove that adding PGua into the perovskite precursor solution retards the crystallization of the perovskite, due to the strong Pb–N bonds, resulting in homogeneous perovskite films with large grains. The PGua-treated films show excellent optoelectronic properties, reduced non-radiative recombination, and prolonged carrier lifetimes. DFT calculations rationalize the improved performance by suppression of surface defect states when adding PGua. Overall, this results in improved device performance and stability, with the *V*_OC_ × FF product reaching unprecedented 85.5% of the Shockley–Queisser limit at given band gap by the synergy of additive engineering with PGua and interfacial passivation treatment with CEAI. Ultimately, we proved the universality of PGua as an additive for multiple device architectures and perovskite compositions. The improved control of perovskite nucleation and morphology from solution by use of PGua as the additive is expected to possess infinite potential for perovskite-based optoelectronic applications such as light-emitting diodes, photodetectors, and resistive memory devices as well as photovoltaics.

## Data availability

The data that support the plots within this paper and other finding of this study are available from the corresponding author upon reasonable request.

## Author contributions

B. Y. and J. S. conceived the idea, fabricated perovskite films and n–i–p devices, and performed characterizations. D. B. carried out the PL mapping measurement, and analyzed the *JV* losses and PL results. W. K., E. M., A. A. A., F. D. A. performed the DFT calculations. C. B. analyzed the data from the PLQY measurements. O. E.-R. measured the fluence-dependent TRPL. G. L. manufactured the p–i–n cells and measured them. J. S., B. Y., D. B., W. K., M. K., U. W. and A. H. participated in editing the manuscript. A. H. directed the overall research. All authors read and commented on the manuscript.

## Conflicts of interest

Authors declare that they have no competing interests.

## Supplementary Material

EE-017-D3EE02344C-s001
