# Supplementary material for: A universal ligand for lead coordination and tailored crystal growth in perovskite solar cells
Source: Energy Environ Sci. 2024 Jan 9;17(4):1549–58. doi: 10.1039/d3ee02344c (PMC10877579; doi:10.1039/d3ee02344c)
Supplement: EE-017-D3EE02344C-s001 [file EE-017-D3EE02344C-s001.pdf]

Electronic Supplementary Material (ESI) for Energy & Environmental Science.
This journal is © The Royal Society of Chemistry 2024

Supplemental Information

Universal ligand for lead coordination and tailored crystal growth in perovskite solar cells

Bowen Yang,[1,2†] Jiajia Suo,[1,2†*] Dmitry Bogachuk,[3,&] Waldemar Kaiser,[4] Clemens Baretzky,[3,5] Oussama Er-Raji,[3,6] Georgios Loukeris,[3,5] Asma A. Alothman,[7] Edoardo Mosconi,[4,7] Markus Kohlstädt,[3,5] Uli Würfel,[3,5] Filippo De Angelis,[4,8,9] Anders Hagfeldt[1,2*]

[1]Department of Chemistry - Ångström Laboratory, Uppsala University, SE-75120 Uppsala, Sweden

[2]Laboratory of Photomolecular Science, Institute of Chemical Sciences and Engineering, School of Basic Sciences, Ecole Polytechnique Fédérale de Lausanne, CH-1015 Lausanne, Switzerland

[3]Fraunhofer Institute for Solar Energy Systems ISE, Heidenhofstr. 2, 79110 Freiburg, Germany

[4]Computational Laboratory for Hybrid/Organic Photovoltaics (CLHYO), Istituto CNR di Scienze e Tecnologie Chimiche "Giulio Natta" (CNR-SCITEC), Via Elce di Sotto 8, 06123 Perugia, Italy

[5]Freiburg Materials Research Center FMF, University of Freiburg, Stefan-Meier-Str. 21, 79104 Freiburg, Germany

[6]Department of Sustainable Systems Engineering (INATECH), Albert-Ludwigs-Universität Freiburg, Emmy-Noether-str. 2, 79110 Freiburg, Germany

[7]Chemistry Department, College of Science, King Saud University, Riyadh 11451, Kingdom of Saudi Arabia

[8]Department of Chemistry, Biology and Biotechnology, University of Perugia, Via Elce di Sotto 8, 06123 Perugia, Italy

[9]SKKU Institute of Energy Science and Technology (SIEST) Sungkyunkwan University, 440-746 Suwon, Korea.

[&] Current address: Solarlab Aiko Europe GmbH, Berliner Allee 29, 79110 Freiburg, Germany

[*] Correspondence: jiajia.suo@kemi.uu.se, anders.hagfeldt@uu.se

[†]Both authors contributed equally to this work.

EXPERIMENTAL PROCEDURES

Synthesis of PGua

Aniline (1.86 g, 20.0 mmol, Sigma-Aldrich) was dissolved in hydrochloric acid solution (36.5% in water, 4 mL), cyanamide (2.1 g, 50 mmol, Sigma-Aldrich) was added into the solution, then the mixture was heated to 85 °C for 12h. Na_2CO_3 solution (10% in water, 20 mL) was dropped into the mixture, a white solid was precipitated. The solid was filtered to get phenylguanidine (2.2 g, 81 % yield) with further purification.

Substrate

Fluorine doped tin oxide (FTO) substrates (NSG-10) were chemically etched by zinc powder and 4 M HCl solution and sonicated in 2% Hellmanex water solution for 30 min, acetone for 15 min and ethanol for 15 min, respectively. Then, all substrates were further cleaned by UV-Ozone for 15 min. Then, a compact TiO_2 layer was deposited on cleaned FTO substrates via spray pyrolysis deposition from a precursor solution of titanium diisopropoxide bis(acetylacetonate) (Sigma-Aldrich) in anhydrous ethanol (Acros), with oxygen as carrier gas. Substrates were heated at 450 °C and kept at this temperature for 15 min before and 30 min after the spray of the precursor solution, then left to cool down to room temperature. Mesoporous TiO_2 layer was spin-coated at 4000 rpm for 20 s, with the acceleration rate of 2000 rpm/s, using a 30 nm TiO_2 paste (Dyesol 30 NR-D) diluted in ethanol with 1:6 volume ratio. After the spin-coating, the substrates were dried at 80 ℃ for 10 min and then sintered at 450 °C for 30 min under dry air flow.

Perovskite layer

The perovskite precursor solution was prepared by dissolving a mixture of cesium iodide (0.075 mmol, TCI Co. Ltd.), methylammonium bromide (0.15 mmol, Dyenamo), formamidinium iodide (1.1275 mmol, Dyenamo), lead iodide (1.575 mmol, Alfa Co. Ltd.) in 1 mL mixture of DMF and DMSO (DMF:DMSO=4:1 v/v, Acros). For the $MAPbI_3·0.05PbI_2$ perovskite precursor, methylammonium iodide (1.4 mmol, Dyenamo), lead iodide (1.47 mmol, Dyenamo) were dissolved in 1 mL mixture of DMF and DMSO (DMF:DMSO=4:1 v/v, Acros). For the $MAPbBr_3·0.05PbBr_2$ perovskite precursor, methylammonium bromide (1.4 mmol, Dyenamo), lead bromide (1.47 mmol, Dyenamo) were dissolved in 1 mL DMSO (Acros). The PGua was dissolved in the precursor solution with different concentration. The perovskite solution was spin-coated through two-step program (1000 rpm for 10 s and 6000 rpm for 20 s) with pouring chlorobenzene as an anti-solvent 5s before the end of the second step. Then the substrates were annealed at 100 °C for 40 min in dry air. The MA vapor-treatment was employed to form high-quality $MAPbBr_3·0.05PbBr_2$ perovskite films according to the literature.[1] The CEAI was dissolved in IPA (5 mg/mL) and the solution was spin-coated at 4000 rpm for 20s on the as-prepared perovskite films and dried on a hot plate at 100 °C for 10 min. The substrates were cooled down to room temperature after annealing the perovskite.

Hole transporting layer and Au top contact

A spirofluorene-linked methoxy triphenylamines (spiro-MeOTAD, 99.9%, Xi'an Polymer Light Technology Corp.) solution was deposited by spin coating at 4000 rpm for 20 s, as hole-transporting material. 90 mg spiro-MeOTAD was dissolved in 1 ml chlorobenzene, doped by 20.6 µL bis(trifluoromethylsulfonyl)imide lithium salt solution (LiTFSI, 520 mg/mL in acetonitrile, Sigma-Aldrich), and 35.5 µL 4-*tert*-butylpyridine (tBP, Sigma-Aldrich). PTAA: 10 mg PTAA (Xi'an Polymer Light Technology Inc) was dissolved in 1mL, doped by 1.6 µL bis(trifluoromethylsulfonyl)imide lithium salt solution (520 mg/mL LiTFSI in acetonitrile, Sigma-Aldrich) and 2 µL 4-*tert*-butylpyridine. Finally, 80 nm of Au top electrode was deposited through thermal evaporator under high vacuum ($< 5*10^{-4}$ Pa) with an active area of 0.16 cm^2.

Preparation of triple cation perovskite precursor for p-i-n PSCs:

For 1.45M of perovskite precursor solution, Cesium Iodide (Sigma-Aldrich), Methylamonium Bromide (Dyenamo), Formamidinium Iodide (Dyenamo) and Lead Iodide (PbI_2, TCI) are being weighed in one vial. The final stoichiometry corresponds to $Cs_{0.05}FA_{0.85}MA_{0.10}Pb(I_{2.9}Br_{0.1})$ with 5% of PbI_2 excess. DMF (Sigma-Aldrich) and DMSO (Sigma-Aldrich) used as solvents with ratio is 4:1. The precursor is stirred for 1 hour at 40°C. After the complete solution of the precursor 0.25 mg of PGua is added and left to stir until the precursor is deposited.

Preparation of double cation wide band gap perovskite precursor for p-i-n PSCs:

For 1.2M of perovskite precursor solution, Cesium Iodide (Sigma-Aldrich), Formamidinium Iodide (Dyenamo), Lead Iodide (TCI) and Lead Bromide (TCI) are being weighed in one vial. The final stoichiometry corresponds to $Cs_{0.17}FA_{0.83}Pb(I_{1.8}Br_{1.2})$. DMF (Sigma-Aldrich) and DMSO (Sigma-Aldrich) were used as solvents with ratio DMF:DMSO 4:1. The precursor is stirred for 2 hours at 40°C. Afterwards, the precursor is being filter with a 0.22μm filter pores and then 1-0.5 mg of PGua is added and left to stir until the precursor is deposited.

Stack preparation for p-i-n PSCs:

The substrates, sputtered with Indium Tin Oxide (ITO) were cleaned with Ethanol, Isopropanol and Water sequentially for 5 minutes each. After drying them, they are processed in an UV-O3 chamber for 20 minutes. [2-(3,6-Dimethoxy-9*H*-carbazol-9-yl)ethyl]phosphonic Acid (TCI) is statically spin coated on the substrate with 3000rpm for 30 seconds and then the substrates are annealed for 10 minutes at 100°C. For the *triple cation* perovskite deposition, a two-step static spin coating program is used. The first step lasts 10 seconds and rotates with 2000 rpm, while the second step utilizes 6000 rpms for 20 seconds. 5 seconds before the end of the second step 150μl of Chlorobenzene are used as antisolvent. Then the substrates are annealed at 100°C for 60 minutes. While for the *double cation wide band gap* perovskite deposition a two-step static spin coating program is used. The first step lasts 10 seconds and rotates with 2000 rpm, while the second step utilizes 4000 rpms for 40 seconds. 10 seconds before the end of the second step 200μl of Chlorobenzene are used as antisolvent. Then the substrates are annealed at 100°C for 15 minutes. Phenyl-C61-butyric acid methyl ester (Ossila) in concentration of 10mg/ml in Chloroform is dynamically spin coated at 4000 rpm for 30 seconds. Bathocuproine (Sigma-Aldrich) in concentration of 0.5mg/ml is also dynamically spin coated at 4000 rpm for 60 seconds. Lastly, 100nm of aluminium are being thermally evaporated in a vacuum chamber. For the first 5nm of the material's deposition, a rate of 1nm per second is utilized. After 5nm of the electrode's thickness have been evaporated, the deposition rate is increased to 10nm per second and kept constant until the end of the process.

Devices Characterization

The solar cell devices were measured using a 300 W Xenon light source (Oriel). The spectral mismatch between AM 1.5 G and the solar simulator was adjusted by a Schott K113 Tempax filter (Prazosopms Gas & Optik GmbH). The light intensity was calibrated with a silicon photodiode with an IR-cutoff filter (KG2, Schott). Current-voltage characteristics were applied by an external voltage bias while measuring the corresponding current with Keithley 2400 source meter in ambient air. The voltage scan rate was 100 mV/s. The devices were covered with a black metal mask with an active area of 0.16 cm^2. ^1H NMR measurements were performed on Bruker AvanceIII-400 MHz NMR spectrometer. Incident photon to current efficiency (IPCE) was carried by a commercial apparatus (Aekeo-Ariadne, Cicci Research s.r.l.). The top-view and cross-section morphologies of the samples was characterized using a high-resolution scanning electron microscope (Zeiss Merlin) with an in-lens secondary electron detector. The operational stability of the devices was measured under a white light-emitting diode lamp with biologic MPG2 potentiostat under N_2 gas flow at maximum power point tracking (MPPT). The thermal test was carried out on a hotplate under N_2 gas flow in the dark.

PL mapping and PLQY measurement

The PL images were optained by OLYMPUS BX50 stereomicroscope and sCMOS camera ("Zyla 5.5 sCMOS" by Andor) with a long-pass filter while the partial illumination of the sample was provided by a 623 nm red light-emitting diode (Thorlabs, SOLIS-623C). Photoluminescence quantum yield (PLQY) measurements were performed using an absolute photoluminescence characterization setup from Quantum Yield Berlin (QYB). The sample was radiated with 532 nm laser light at different photon fluxes (equivalent to 0.007-10 Suns) focused on a sample (placed in an integrating sphere) with an illumination spot size of 0.1 cm2. The measured counts were obtained with an integration time of 0.5 s and averaged over 10 spectra.

TrPL measurement

Transient photoluminescence measurements were performed with a UV-vis photomultiplier tube and a single-photon counting device (Timeharp). A 515 nm laser (Omicron) was used as a light source and it was modulated digitally with a trigger signal generated by an arbitrary wave-form generator. The laser power during on-time was adjusted to match the Jsc of a perovskite solar cell under 1-sun illumination (AM 1.5G) and lower intensities. The on-time was set to 240 μs and the off-time to 10 μs. The integration time was set to 300 s. The spot size was around 0.785 cm^2.

XRD measurement

The X-ray diffraction patterns were recorded with PANalytical Empyrean system with a PIXcel-1D detector, Bragg-Brentano beam optics and parallel beam optics. Light source is from copper Kα beam filtered with nickel β filter. Diffraction spectra were characterized between 2-theta of 5° and 50° at a scan rate of 1° per minute with the step width of 0.02^o

Other measurements

^{1}H NMR measurements were performed on Bruker AvanceIII-400 MHz NMR spectrometer. XPS measurements were carried out on an Axis Supra apparatus (Kratos Analytical) using the monochromated Kα X-ray line of an aluminum anode. The pass energy was set to 20 eV with a step size of 0.1 eV. Scanning electron microscopy was performed on a ZEISS Merlin high-resolution (HR)-SEM.

Table S1: Additive binding energy with PbI_2 precursor complex.

**Additive**	**E_{rel} (eV)**	**E_{rel} - $E_{rel,PGua}$ (eV)**
**PGua**	**-1.49**	**0**
Methylamine	-1.32	0.17
Methimazole	-1.14	0.35
N-methyl-2-pyrrolidone	-0.98	0.51
Thiourea	-0.92	0.57
Ethyl acetate	-0.83	0.66
Rhodanine	-0.73	0.76

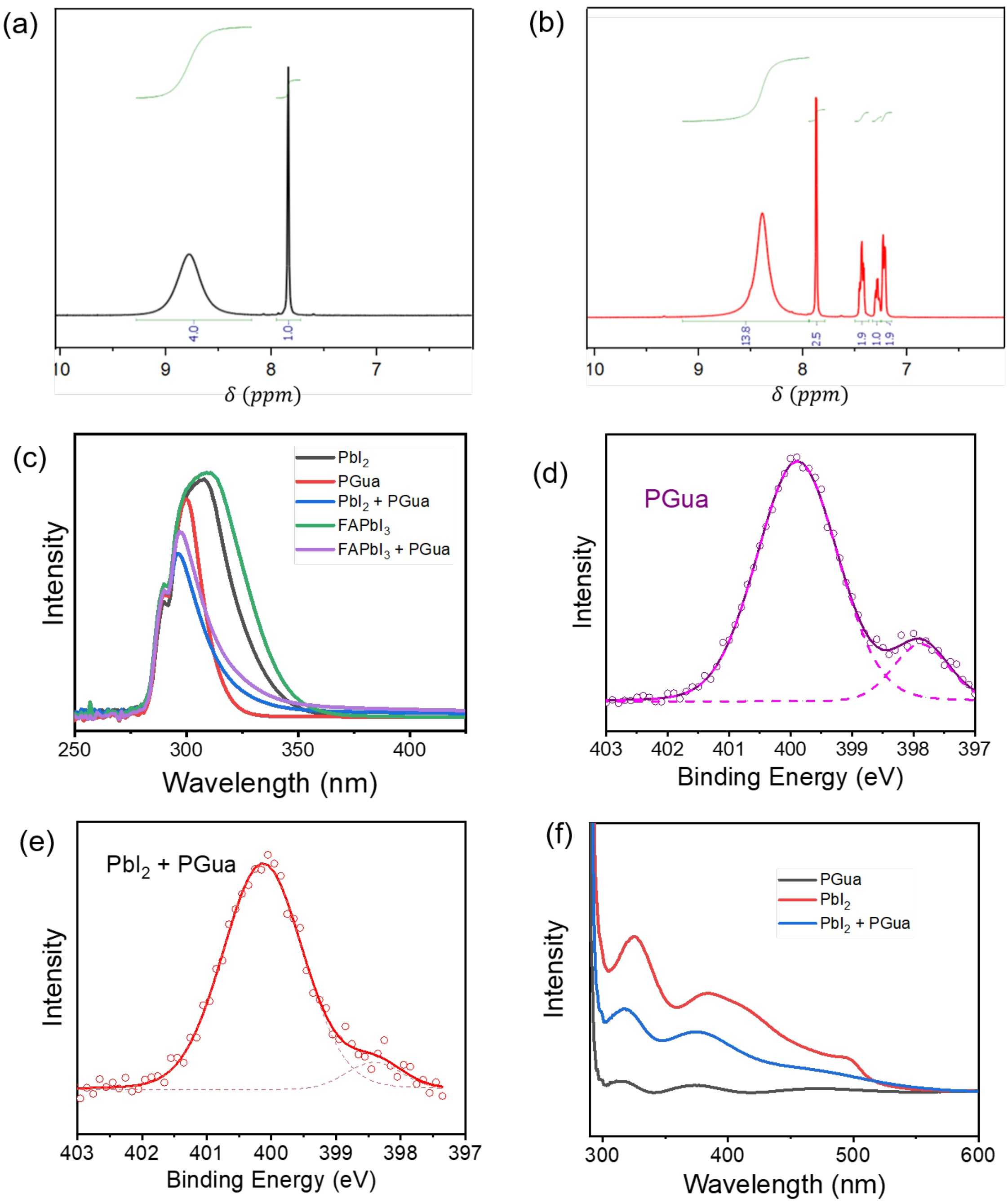

Figure S1. ^{1}H NMR spectra of (a) $FAPbI_3$ solution and (b) the mixed solution of PGua and $FAPbI_3$ (the molar ratio of PGua and $FAPbI_3$ is 1:2.5), which were dissolved in DMSO-*d6*. The integration of peaks shows the NH peak from guanidine is mixed with the NH peak from FA^+. (c) The UV-Vis absorption of PbI_2, PGua, the mixture of PbI_2 with PGua, $FAPbI_3$ and the mixture of $FAPbI_3$ with PGua solution, which were dissolved in DMSO solution with the concentration of 0.3 mM. XPS core level signals of N 1s of PGua (d) and the mixture of PbI_2 with PGua (e). (f) The UV-Vis absorption of PbI_2, PGua, the mixture of PbI_2 with PGua film. The molar ratio of PGua and PbI_2 ($FAPbI_3$) is 1:1 in the above experiments (c-f).

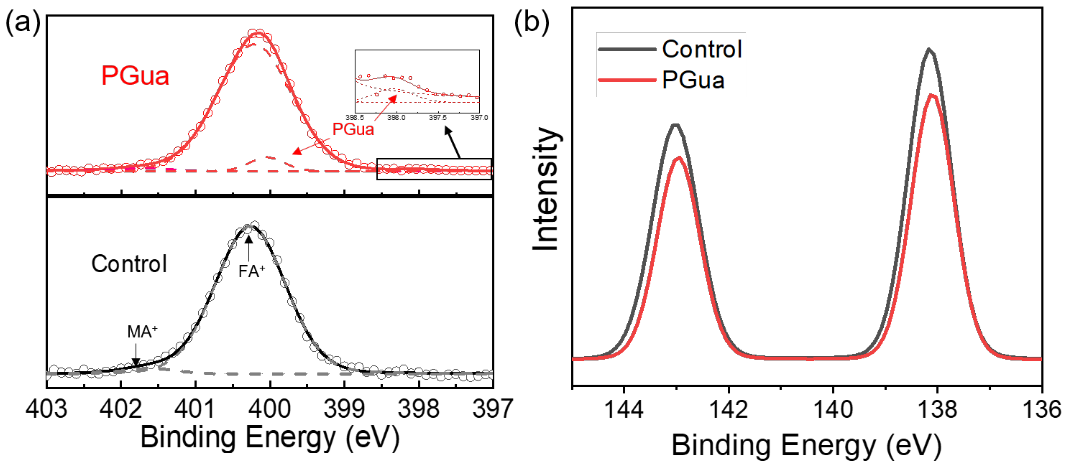

Figure S2. XPS core level signals of (a) N1s and (b) Pb4f of reference perovskite film (black) and PGua treated perovskite film (red).

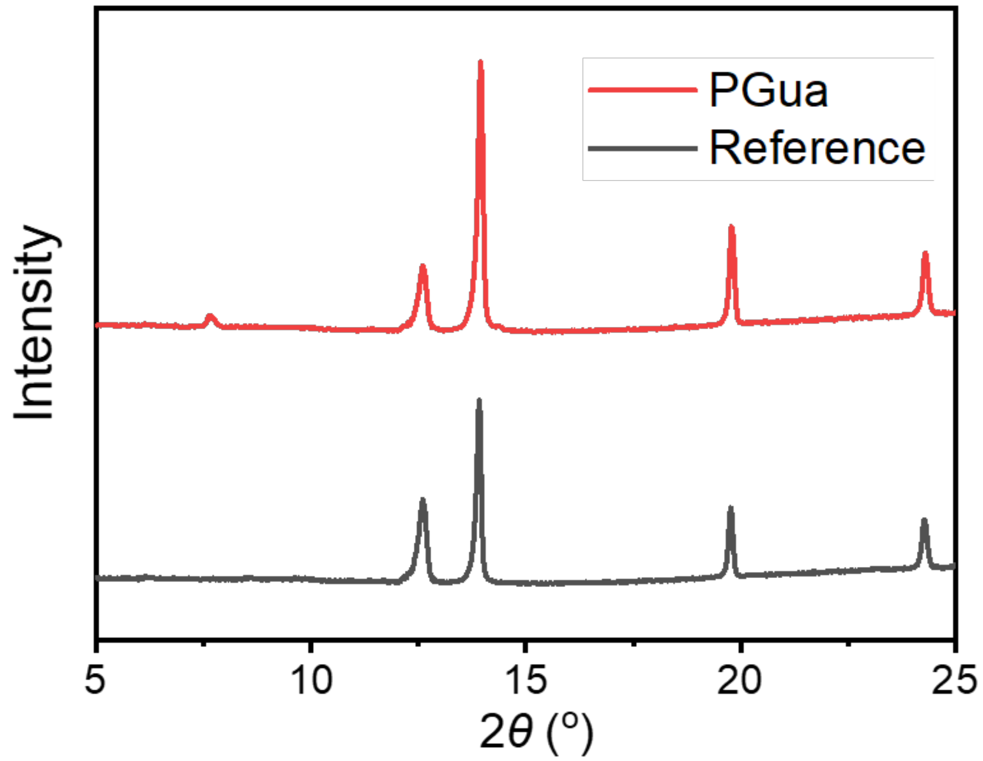

Figure S3. XRD patterns of reference perovskite film (black) and PGua doped perovskite film (red), the peaks intensity ratio of PbI_2 (12.6°) and 3D perovskite (13.9°) is 1:2 and 1:3.6, respectively.

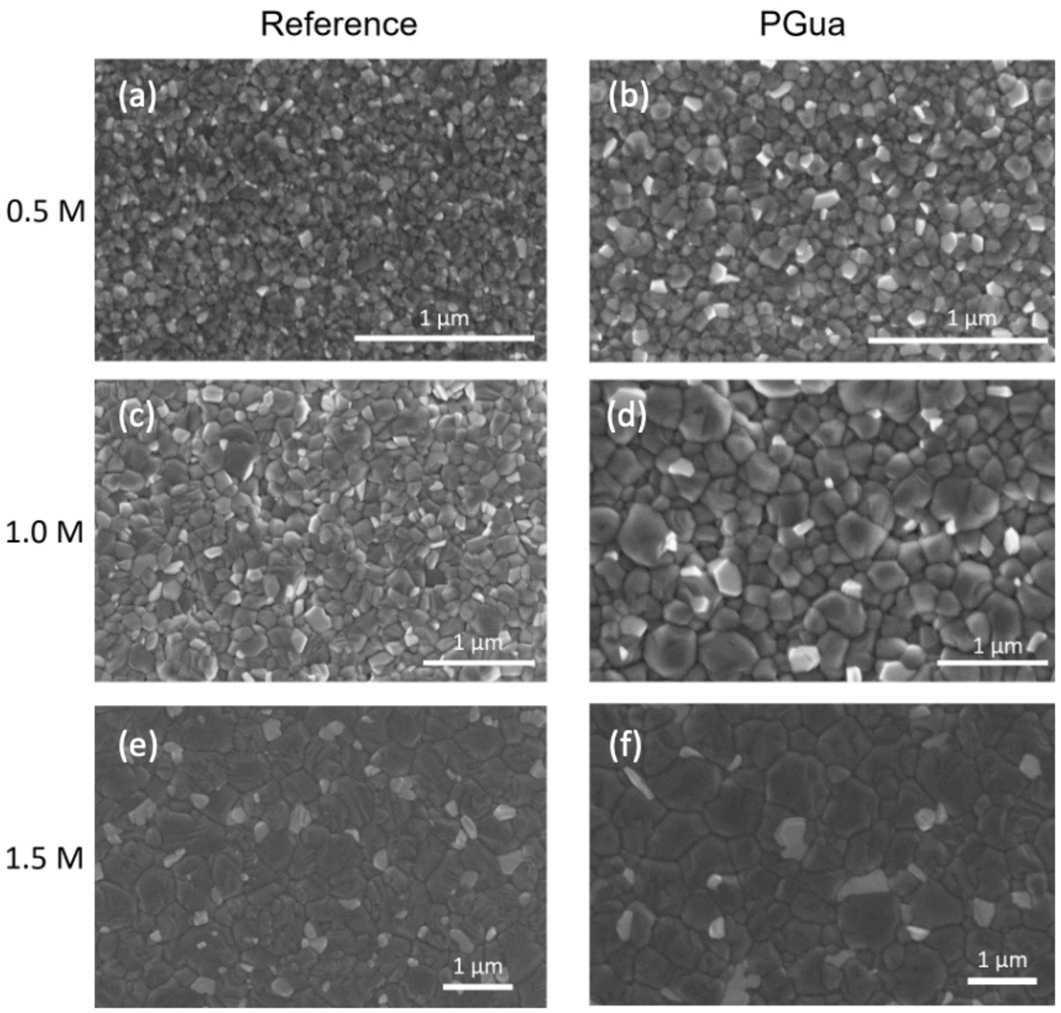

Figure S4. Top-view SEM images of reference perovskite films (a, c, e) and PGua treated perovskite films (b, d, f) with different concentrations of precursor solutions, (a, b) 0.5 M, (c, d) 1.0 M, (e, f) 1.5 M, respectively.

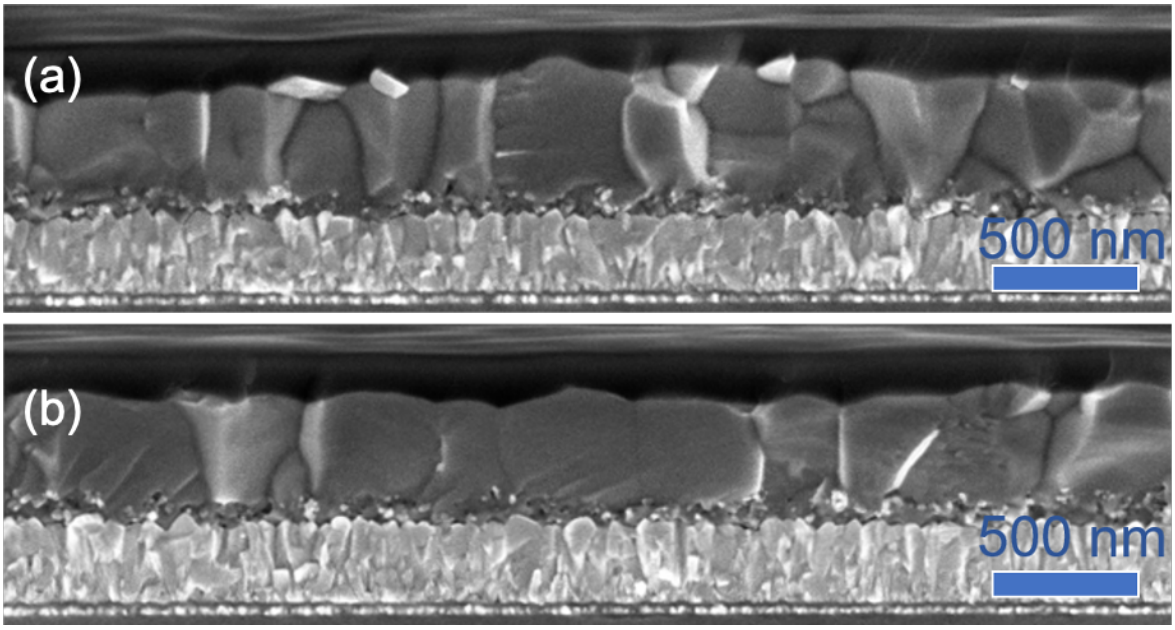

Figure S5. Cross-Sectional SEM images of (a) reference perovskite film and (b) PGua-treated perovskite film.

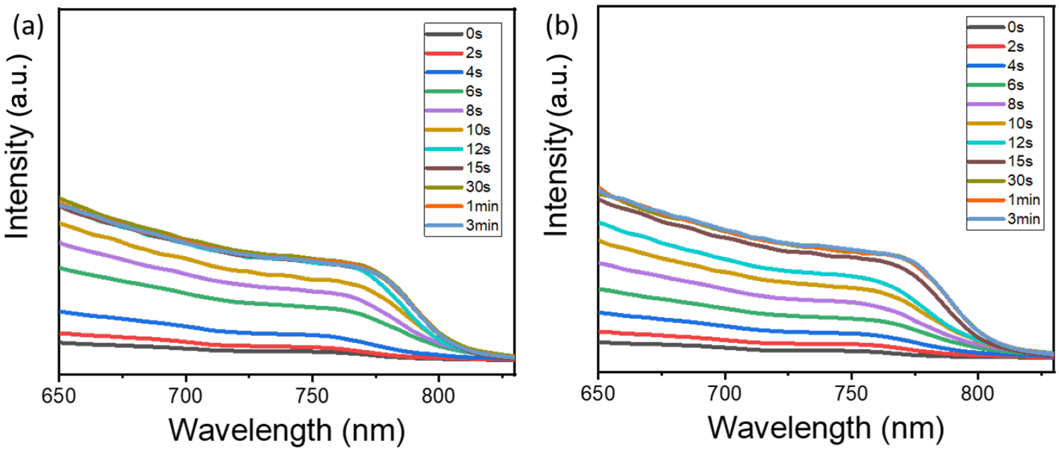

Figure S6. UV-vis absorption spectra of (a) reference perovskite film and (b) PGua-treated perovskite film at various annealing times.

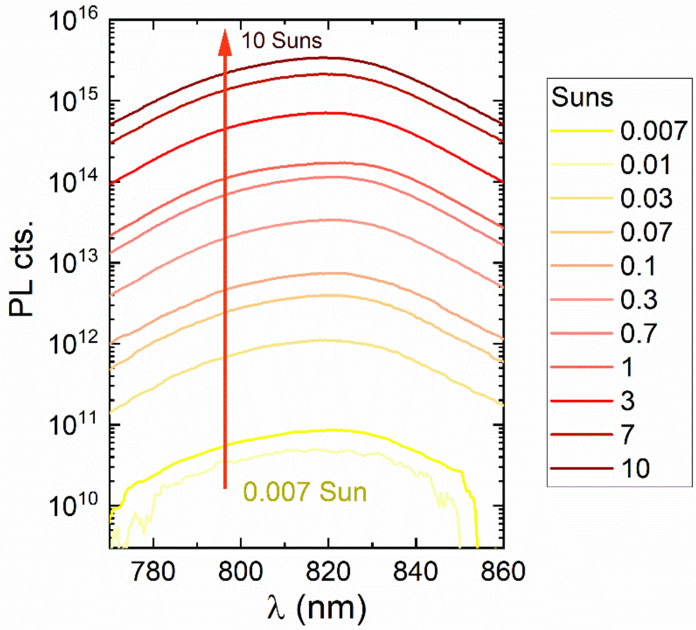

Figure S7. Absolute PL spectra of perovskite films on glass under different incident illumination intensities.

Supplementary Note 1

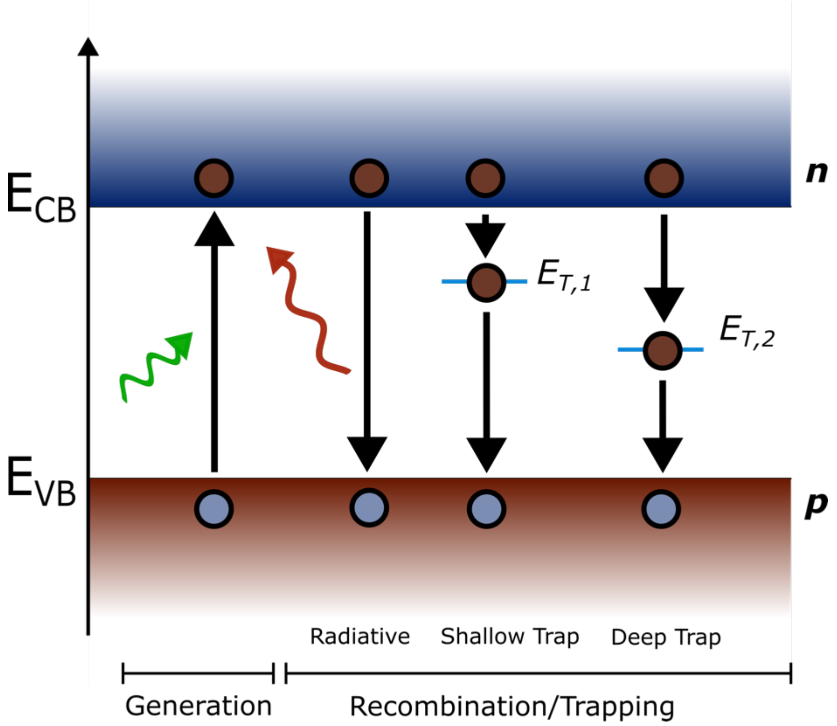

Figure S8. Generation and Recombination pathways in a two-trap level SRH model.

The rate equation to describe the intensity dependent change in charge carrier density was modeled with generation, radiative recombination and a two-trap level Shockley-Read-Hall mechanism as depicted in **Figure S8**. While radiative recombination is described as classical band to band recombination, two nonradiative recombination pathways are mediated by trap states.

The recombination of charge carriers (electrons n and holes p) via trap states, known as the Shockley-Read-Hall (SRH) mechanism, is governed by the following equation:

$$R_{\mathrm{SRH}} = \frac{np - n_i^2}{(n + n_1)\tau_p + (p + p_1)\tau_n}$$

Therein, n_i^2 is the square of intrinsic charge carrier concentration The quantities $n_1 = N_c \exp\left(\frac{E_t - E_c}{kT}\right)$ and $p_1 = N_v \exp\left(\frac{E_v - E_t}{kT}\right)$ represent the concentrations of electrons and holes in the trap states, which are functions of the relative trap energy level E_t and temperature T, with k being the Boltzmann constant. Furthermore, the so called carrier lifetimes $\tau_n = \frac{1}{N_t \sigma_n v_{th}}$ and $\tau_p = \frac{1}{N_t \sigma_p v_{th}}$ correspond to the minority carrier lifetimes for electrons and holes, which depend on the product of trap density N_t, capture cross section σ_n of the respective charge carrier and the thermal velocity v_{th}.

If the electron and hole concentrations are about equal ($n = p$) and significantly exceed the intrinsic carrier concentration (n_i), the SRH recombination rate simplifies to:

$$R_{\mathrm{SRH}} = \frac{n^2}{(n + n_1)\tau_p + (n + p_1)\tau_n}$$

For shallow traps it follows that n_1 >> p_1 or p_1 >> n_1 dependent on whether the trap energy is close to the conduction band or the valence band. For low generation rates it can also be assumed that n << p_1 or n << n_1. Thus, as either n_1 or p_1 govern the denominator of the SRH recombination rate, we only must consider either τ_p (if n_1 >> p_1) or τ_n (if p_1 >> n_1). For simplicity we therefore set $\tau_p = \tau_n$.

Considering two independent SRH recombination mechanisms, generation and radiative recombination leads to the following equation for steady-state conditions:

$$0 = G - n^2 k_r - R_{\mathrm{SRH,E1}} - R_{\mathrm{SRH,E2}}$$

Where $R_{\mathrm{SRH,E1}}$ is the SRH recombination rate via a trap level at energy ET,1 and $R_{\mathrm{SRH,E2}}$ is the SRH recombination via a second trap level with energy ET,2. The other parameters are the generation rate G and k_r as the external radiative recombination coefficient, respectively.

The steady state PLQY was then calculated as

$$PLQY_{calc,G} = k_r n^2/G$$

Therein, G is the averaged generation rate calculated from the photon flux density divided by the layer thickness of 470 nm. An effective density of states of $N_c = N_v = 2.0\ 10^{18}$ cm^{-3} was assumed.[1] The external radiative recombination coefficient was set to $k_{r,\mathrm{ext}} = 10^{-13}$ cm^3s^{-1}. The energy level of the second trap level T_2 is set to be approximative mid-bandgap $E_{T,2} = -4.75$ (eV) between the conduction band edge E_c =-4.0 (eV) and the valence band edge E_V =-5.53 (eV)

The free parameters from the steady-state equation were chosen to minimize the squared difference as

$$S = \sum_G \left\| PLQY_{measured,G} - PLQY_{calc,G} \right\|^2$$

for the different generation rates G used for the intensity dependent PLQY measurements.

	PGua modified sample	Reference sample
$\tau_{p,1/n,1}$ (s)	2.32^-6	1.53^-6
$\tau_{p,2/n,2}$ (s)	2.34^-5	1.75^-5
ET,1 (eV)	-4.0269	-4.0643

Supplementary Note 2

The data from the transient photoluminescence (TRPL) was normalized and fitted with the bi-exponential decay formula

$$I(t) = I_0 \exp\left(-\frac{t}{\tau_0}\right) + I_1 \exp\left(-\frac{t}{\tau_1}\right)$$

With the normalized overall count rate $I,$ the two initial Intensities I_0 and $I_1$and the two lifetimes τ_0 and τ_1.

Using a linear loss function

$$S = \left\| I_{measured,TRPL} - I_{bi-exp} \right\|^1$$

leads to an good approximation of the measured data except for the TRPL-Data of the PGua sample measured at 1 Sun equivalent fluence. Using a root loss function

$$S = \left\| I_{measured,TRPL} - I_{bi-exp} \right\|^{1/2}$$

increases the fit quality for the that sample but decreases the perceived fit quality with most other measurements.

The differential lifetime τ_{TPL}

$$\tau_{\mathrm{TPL}} = \left(-\frac{1}{m}\frac{d\ln(\phi_{\mathrm{TPL}})}{dt}\right)^{-1}$$

$$\tau_{\mathrm{TPL}}/m = -\left(\frac{d\ln(\phi_{\mathrm{TPL}})}{dt}\right)^{-1}$$

from the bi-exponential fit is depicted in **Figure S9** b) and d).

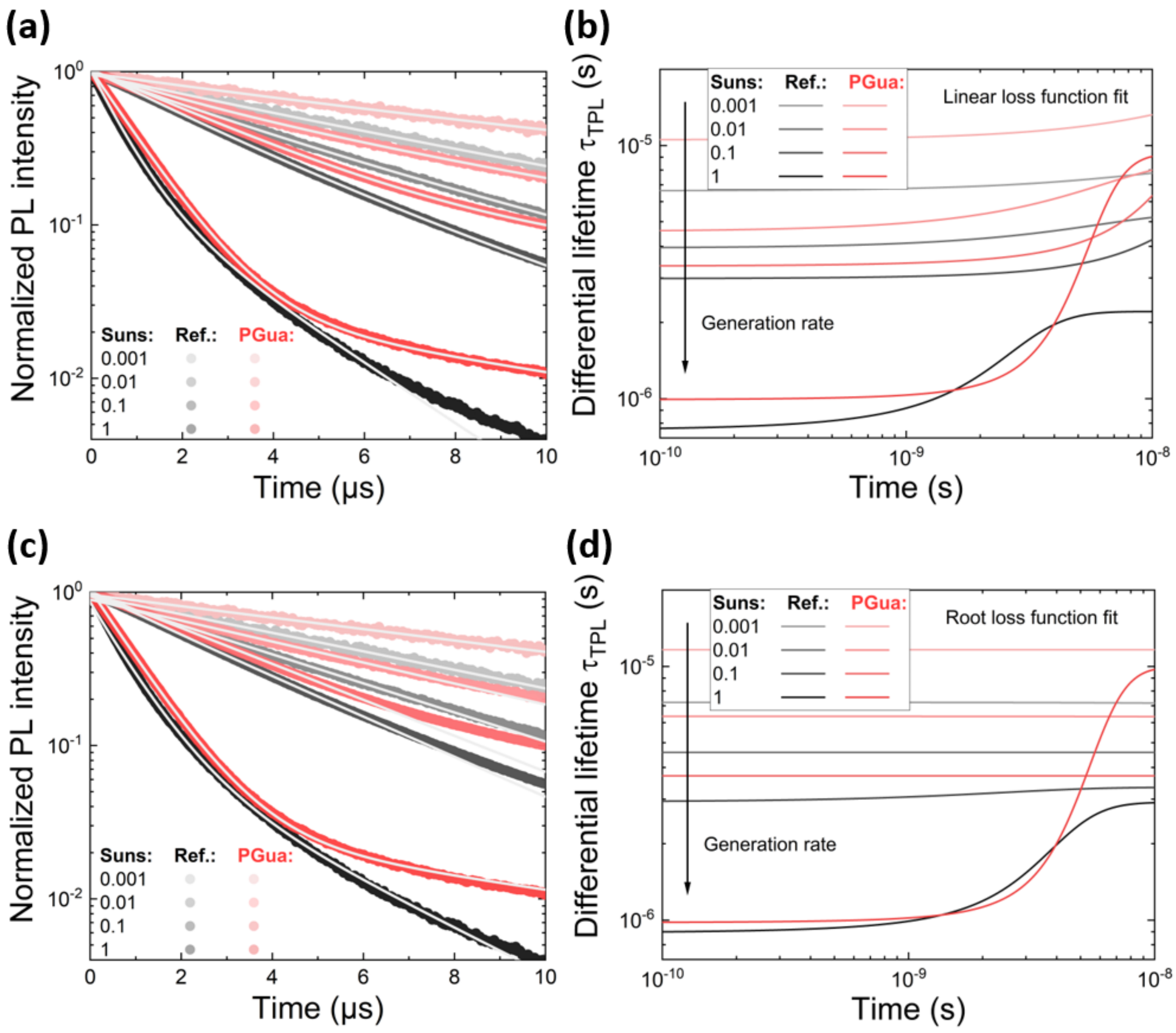

Figure S9. Light intensity dependent, normalized Time-resolved PL (TRPL) decays with corresponding bi-exponential fits for using a linear (a) and square-root (c) loss function for reference perovskite (black) and PGua doped perovskite (red). (b) and (d) show the differential lifetimes extracted from the bi-exponential fit (white lines in (a) and (c)) with the linear and square-root loss function, respectively.

Supplementary Note 3

DFT calculations have been carried out on the (001) $MAPbI_3$ surface within supercell approach by using the Perdew-Burke-Ernzerhof (PBE)[2] functional. Slab models have been built starting from the tetragonal phase of MAPbI3, by fixing cell parameters to the experimental values.[3] This approach has been already applied previously to ensure a proper comparison with the MAPbI3 systems.[4] A 15 Å of vacuum were added along the non-periodic direction perpendicular to the slabs in all cases.

Perovskite models are simulated using the Quantum Espresso package.[5] PBE calculations have been performed by using ultrasoft pseudopotentials (shells explicitly included in calculations: F 2s, 2p; Br 4s, 4p; I 5s, 5p; N, C 2s, 2p; H 1s; Pb 6s, 6p, 5d) and a cutoff on the wavefunctions of 40 Ryd (320 Ryd on the charge density). DFT-D3 correction were also included.[6] Electronic structures of the pristine bulk and the PGua passivated bulk supercells were refined using the hybrid HSE06 exchange correlation functional with α=0.43 and inclusion of spin-orbit coupling corrections. The calculated DOS reported in the diagrams in Figure S9 have been aligned to the respective VB level in all cases.

Starting from the flat PbI_2-terminated (001) surface, PGua molecules were added in the two defective slabs: V_{PbI2} and Iodine Frenkel (V_I^+/I_i^-). The calculation of additives inside the $MAPbI_3$ bulk was carried out on the 2x2x2 tetragonal supercell with a PbI_2 vacancy (V_{PbI2}), with optimized ionic and cell parameters to estimate volume changes.

The formation energy of the PbI_2 vacancy at the surface and in the bulk is given by:

$$E_F(V_{\mathrm{PbI2}}) = E(\mathrm{def.}) + \mu(\mathrm{PbI_2}) - E(\mathrm{prist.}) \quad (1)$$

where $E(\mathrm{prist.})$ is the pristine reference system (bulk or surface), $E(\mathrm{def.})$ is the respective system with the V_{PbI2}, and $\mu(\mathrm{PbI_2})$ is the chemical potential of a PbI_2 unit, obtained from the geometry optimized PbI_2 phase, COD ID 9009114.[7]

The passivation energy when adding PGua (or other additives in the bulk) at the respective surface/bulk defects is calculated as:

$$E_{\mathrm{pass}} = E(\mathrm{PVK + additive}) - E(\mathrm{def.}) - E(\mathrm{additive}) \quad (2)$$

where $E(\mathrm{PVK + additive})$ is the additive-passivated perovskite, and $E(\mathrm{additive})$ is the energy of a single additive molecule.

The formation energy of the iodine Frenkel (V_I^+/I_i^-) defect at the perovskite surface is directly given by:

$$E_F(\mathrm{V}_I^+/\mathrm{I}_{\mathrm{int}}^-) = E(\mathrm{def.}) - E(\mathrm{prist.}) \quad (3)$$

Note that, as all considered defects are charge neutral, no electrostatic corrections are required.

Molecular complexes simulations have been carried out using Gaussian09 program package [8] with the B3LYP functional [9] along with the lanl2dz basis set for Pb and I and 6-31G* for the other species, including empirical DFT-D3 dispersion corrections.[10]

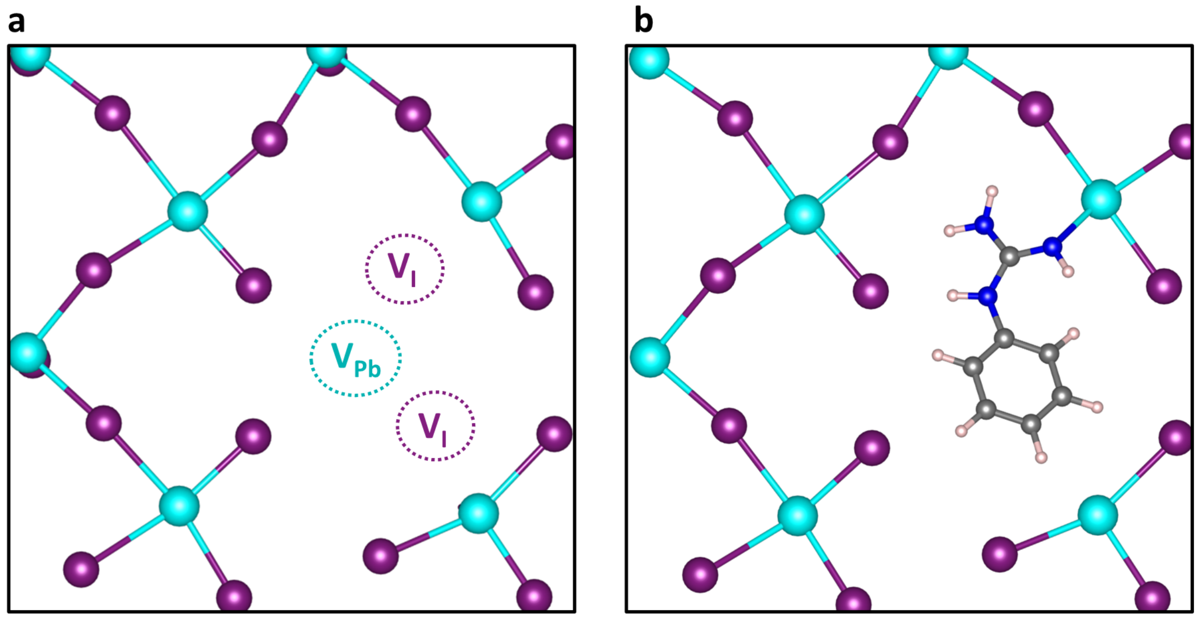

Figure S10. Top view of the PbI_2-terminated surface layer with (a) PbI_2 Schottky defect, V_{PbI2}, and (b) after passivation with PGua. All remaining layers of the slab have been removed for an improved visibility of the Schottky defect.

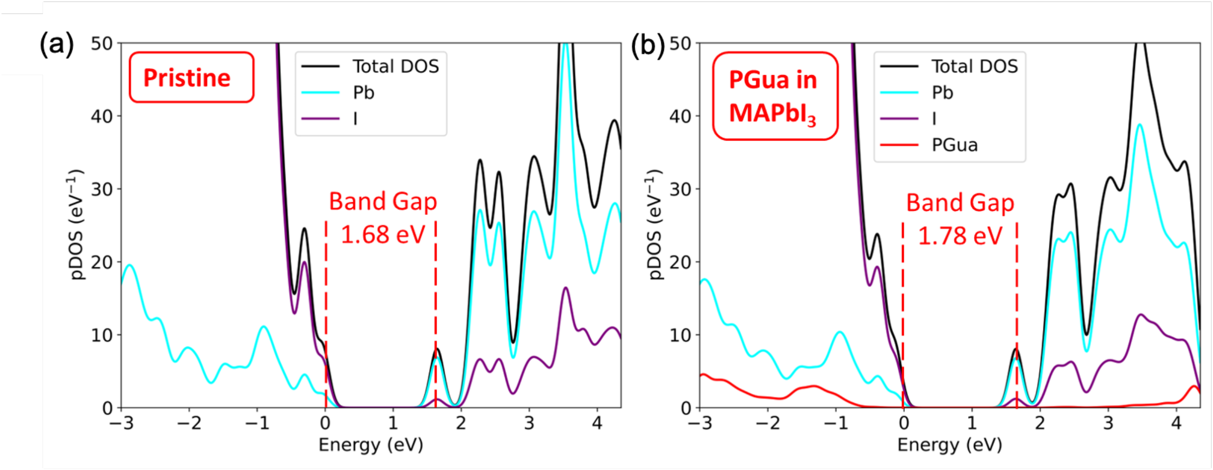

Figure S11. Density of states of (a) pristine and (b) passivated bulk perovskite at the HSE06+SOC level of theory. In (b), PGua passivates a neutral PbI_2 vacancy.

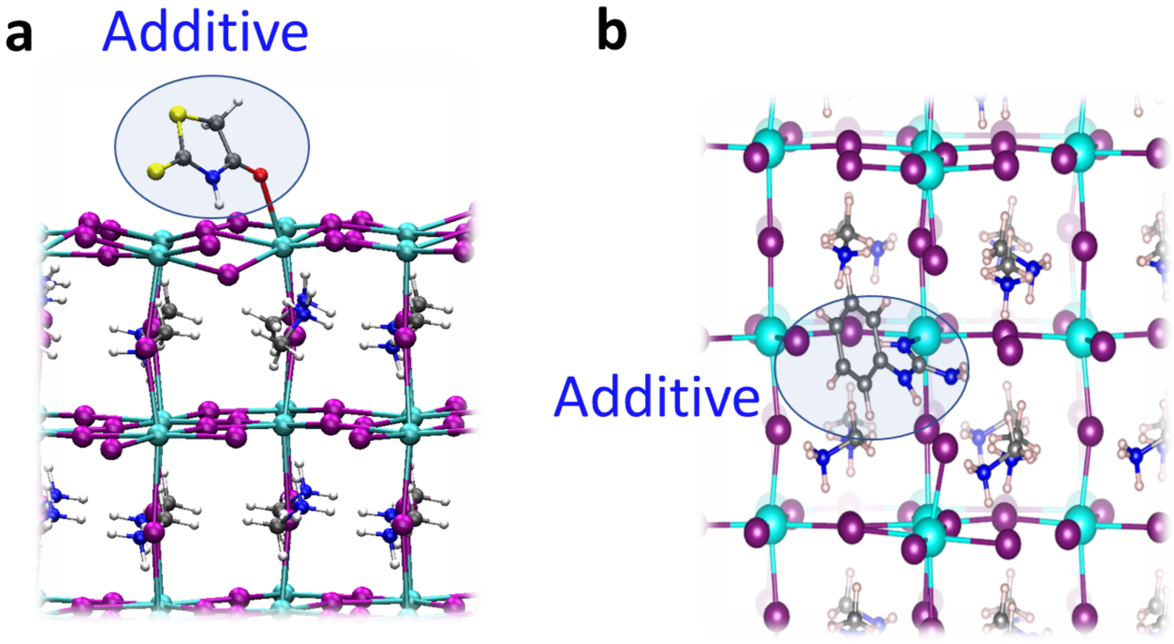

Figure S12. Surface and bulk passivation by additives: (a) Structural representation of an additive molecule bonded to a undercoordinated Pb ion at the PbI_2-terminated surface. (b) $MAPbI_3$ bulk with an additive molecule passivating a PbI_2 vacancy.

Table S2: Additive binding energy with undercoordinated Pb ions at the perovskite grain surface shown in **Figure S10a**.

**Additive**	**E_{pass} (eV)**	**E_{rel} - $E_{pass,PGua}$ (eV)**
**PGua**	**-1.25**	**0**
Methylamine	-1.01	0.25
Methimazole	-1.15	0.10
N-methyl-2-pyrrolidone	-0.87	0.30
Thiourea	-1.26	-0.01
Ethyl acetate	-0.69	0.56
Rhodanine	-1.07	0.19

a. Passivation energy E_{pass} is given eqs. (1, 2).

Table S3: Passivation of PbI_2 vacancies in the $MAPbI_3$ bulk shown in **Figure S10b**.

**Additive**	**$E_{pass}{}^{a}$ (eV)**	**E_{pass}- $E_{pass,PGua}$ (eV)**	**ΔV^{b} (%)**
**PGua**	**-0.76**	**0**	**0.76**
Methylamine	-0.30	0.46	-0.09
Methimazole	-0.45	0.31	0.27
N-methyl-2-pyrrolidone	-0.13	0.63	0.36
Thiourea	-0.65	0.11	0.21
Ethyl acetate	-0.13	0.64	0.24
Rhodanine	-0.48	0.29	0.46

a. Passivation energy E_{pass} is given eqs. (1, 2). *b.* ΔV gives the volume change when passivating a PbI_2 vacancy by the respective additive.

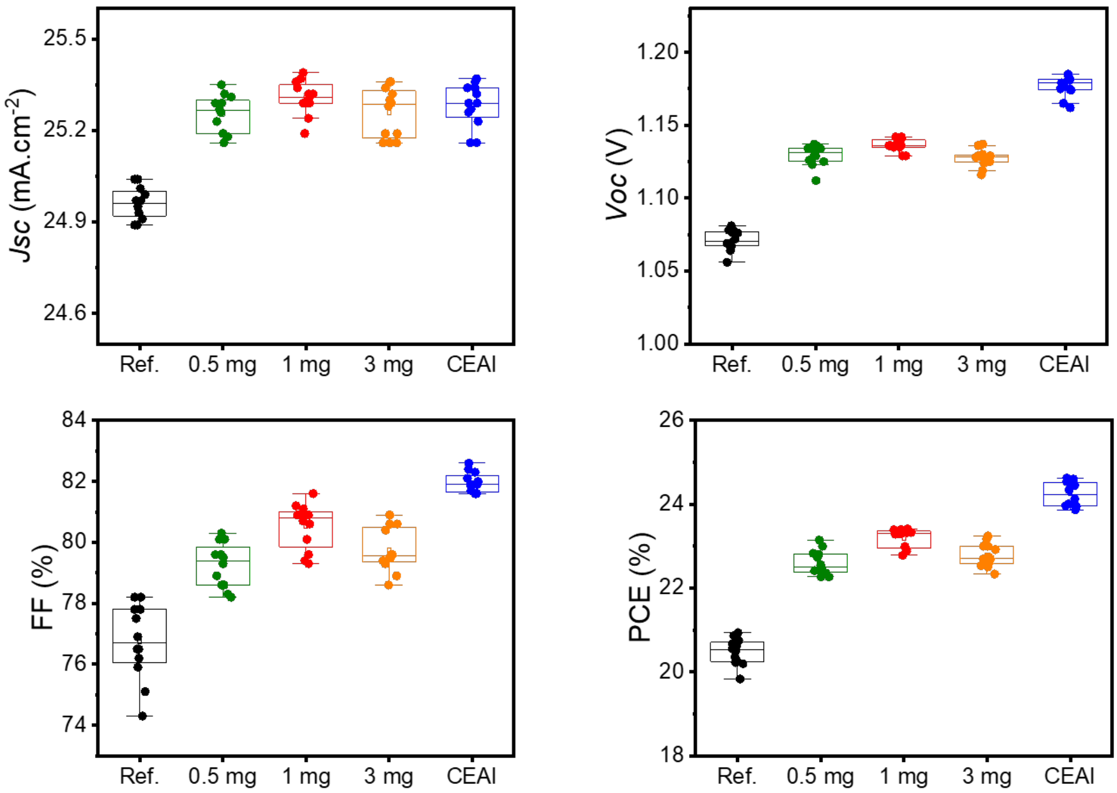

Figure S13. Statistical box charts for the photovoltaic parameters of (a) J_{SC}, (b) V_{OC}, (c) FF, and (d) PCE of the 15 devices each condition without and with different concentrations PGua treatment (0.5 mg, 1.0 mg, 3 mg), CEAI (20 mM) treatment.

Table S4. Champion Photovoltaic Parameters of PSCs with, without PGua dopant and with CEAI treatment.

	Jsc (mA/cm^2)	*Voc* (V)	FF (%)	PCE (%)	HI[c] (%)
Reference RS[a]	24.97	1.078	77.9	20.98	5.3
Reference FS[b]	24.95	1.068	74.6	19.87	
PGua RS	25.29	1.142	81.1	23.41	1.6
PGua FS	25.28	1.136	80.2	23.04	
PGua+CEAI RS	25.37	1.181	82.2	24.62	0.7
PGua+CEAI FS	25.32	1.179	81.9	24.44	

[a]Reverse scan. [b]Forward scan. [c]HI = $(PCE_{RS}-PCE_{FS})/PCE_{RS}$

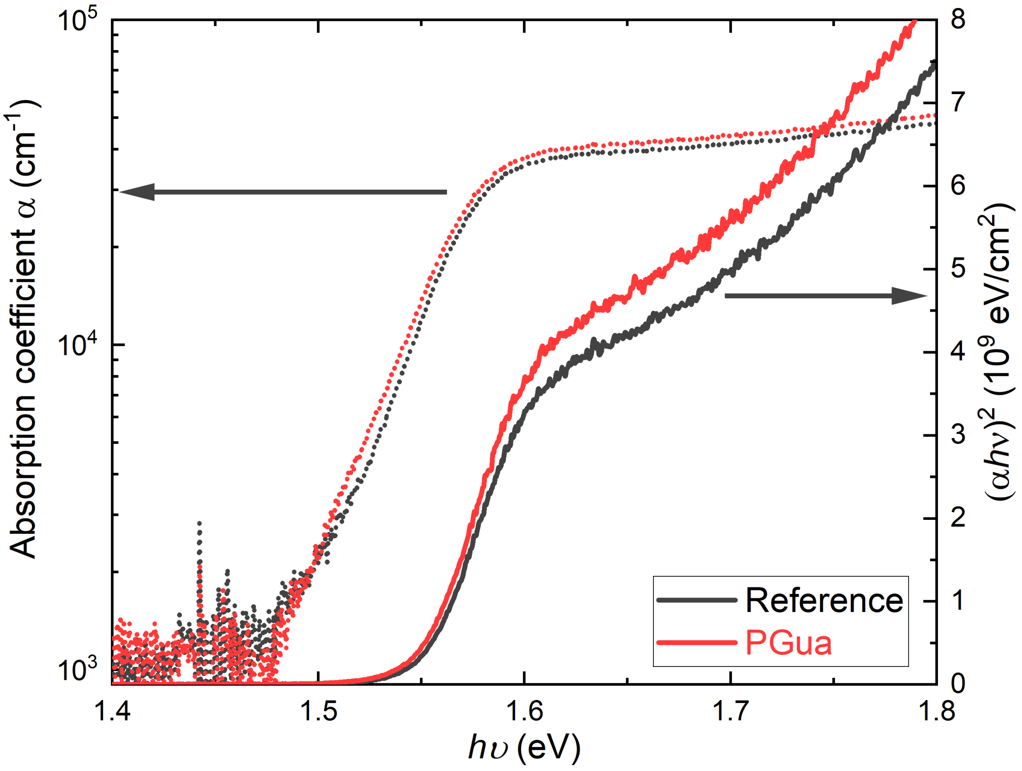

Figure S14. Absorption coefficient measurement and obtained Tauc plot of reference perovskite (black) and PGua doped perovskite films (ref) on glass.

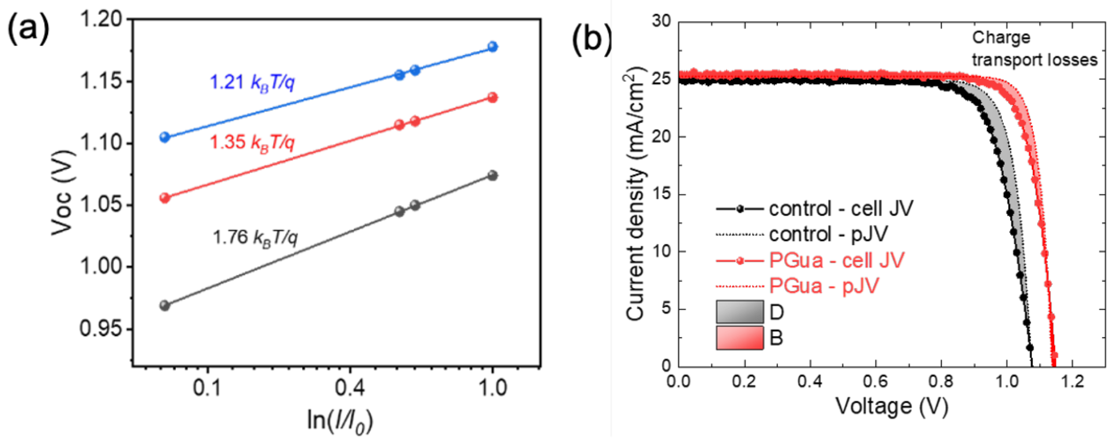

Figure S15. (a) Light intensity-dependent measurement of Voc of reference device (black), PGua treated device (red) and combined CEAI passivated device (blue). (b) cell JV-curves compared with the pseudo JV-curves, reconstructed from **Fig. 4a** of the reference and PGua-passivated perovskite solar cells, where the difference between them represents the charge transport losses.

Supplementary Note 4

		Reference	PGua	PGua+CEAI
Perovskite solar cell	Jsc (mA/cm2)	24.9	25.3	25.4
	Voc (V)	1.078	1.142	1.181
	FF (%)	77.9	81.1	82.2
	PCE (%)	20.98	23.41	24.6
Pseudo JV curve	$pFF^{cell\ (\%)}$	82.9	86.3	88
	$pPCE^{cell\ (\%)}$	22.2	24.8	26.4
Series resistance loss	pFF^{cell} – FF (%)	5	5.2	5.8
	$pPCE^{cell}$ – PCE (%)	1.22	1.4	1.8
Perovskite film	QFLS/q (V)	1.11	1.149	-
	$pFF^{pero\ (\%)}$	86.47	86.73	-
	$pPCE^{pero\ (\%)}$	24	25.2	-
Interfacial non-rad. Rec. loss	QFLS/q-Voc (mV)	35	7	-
	pFF^{pero}-$pFF^{cell\ (\%)}$	3.57	0.39	-
	$pPCE^{pero}$-$pPCE^{cell\ (\%)}$	1.73	0.38	-
Perovskite non-rad. rec. losses (bulk + surface)	$(QFLS_{max}$ -QFLS)/q	0.146	0.11	-
	FF^{SQ}-pFF^{pero}	3.7	3.4	-
Perovskite non-rad. bulk rec. Loss*	PCE^{SQ}-$PCE^{PGua+CEAI}$	5.48	5.48	5.48
Perovskite non-rad. surface rec. Loss*	$PCE^{PGua+CEAI}$ - $pPCE^{pero}$	2.32	1.07	0

Reduction of loss by PGua in comparison to reference	Voc - non-rad. rec. at interfaces (mV)	0	28	-
	FF - non-rad. rec. at interfaces (%)	0	3.18	-
	PCE - non-rad. rec. at interfaces (%)	0	1.35	-
	PCE - perovskite surface recombination (%)	0	1.25	-

Since the Voc of the PSC with PGua and CEAI is even higher than the QFLS of the PGua-passivated perovskite film, we rely on the assumption that the QFLS must be at least 1.181 V. Hence, the remaining 79 mV (or less) difference between the QFLSmax and the QFLS of the PGua+CEAI sample is attributed to an additional non-radiative recombination in the bulk and at perovskite surface. Considering that CEAI is a surface passivation applied as a compact layer on top of the perovskite film, we assume that it cannot significantly alter the bulk non-radiative recombination and is primarily passivating the surface. Hence, for the arbitrary calculation of the PCE losses due to perovskite surface recombination, we consider PGua- and CEAI-passivated sample to be limited only by the non-radiative recombination in the perovskite bulk.

The maximum QFLS of a semiconductor ($QFLS_{max}$) can be found from the material bandgap and absorptivity using the following equation:

$$QFLS_{max} = kT \ln\left(\frac{J_{rad}}{J_{0,rad}}\right) = kT \ln\left(\frac{h^3 c^2}{2\pi} \frac{\int a(E)\Phi_{in} dE}{\int E^2 a(E) \exp\left(\frac{-E}{kT}\right) dE}\right)$$

, where h is Planck constant, c – light speed, a – absorptivity, E – photon energy and Φ_{in} is incident photon flux.

Using the common notion that the $a(E > E_G) = 1$ and $a(E < E_G) = 0$, the equation X can be solved to find the maximum QFLS for a specific E_G. Based on the emission peak from PLQY measurements at 1.53 eV, we calculated the $QFLS_{max}$ of perovskite studied in this work to be 1.26 eV.

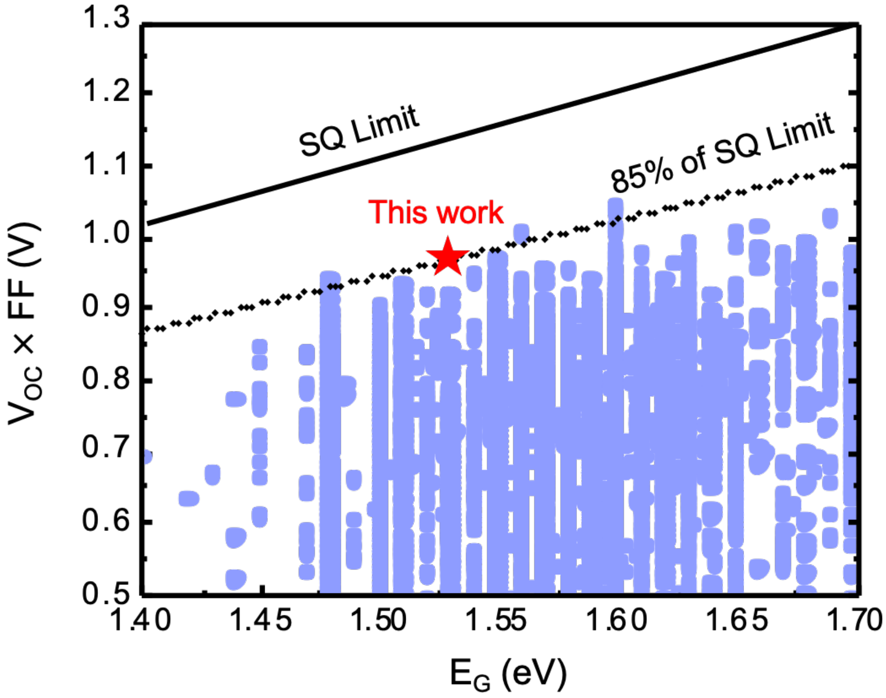

Figure S16. Product of Voc and FF of the manufactured PSCs found in the perovskite database as a function of the absorber bandgap (E_G) in comparison to the champion cell shown in this work. The data of over >28,000 PSCs shown in this plot was gathered from perovskite database.

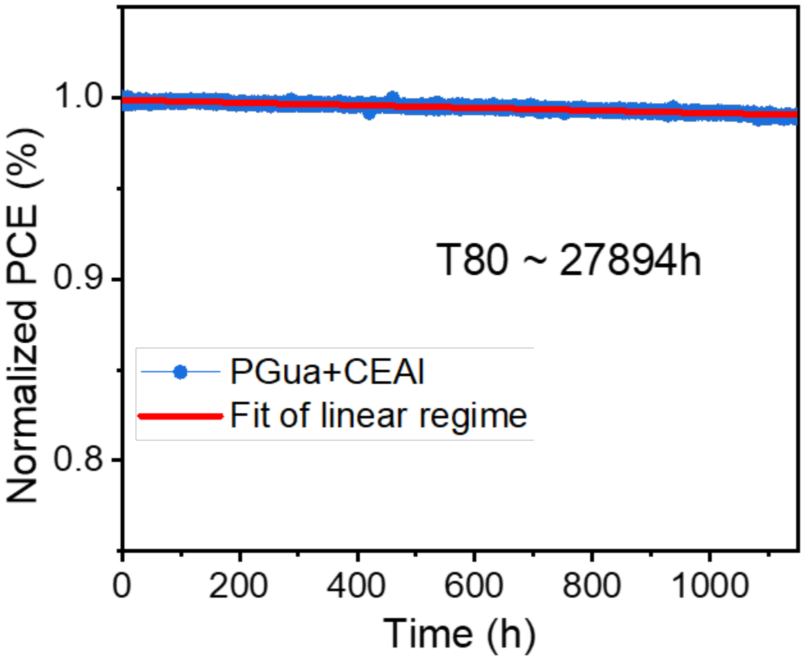

Figure S17. The linear fitting of MPPT of combined passivated device, the slope of the fitting line is $7.17*10^{-6}$.

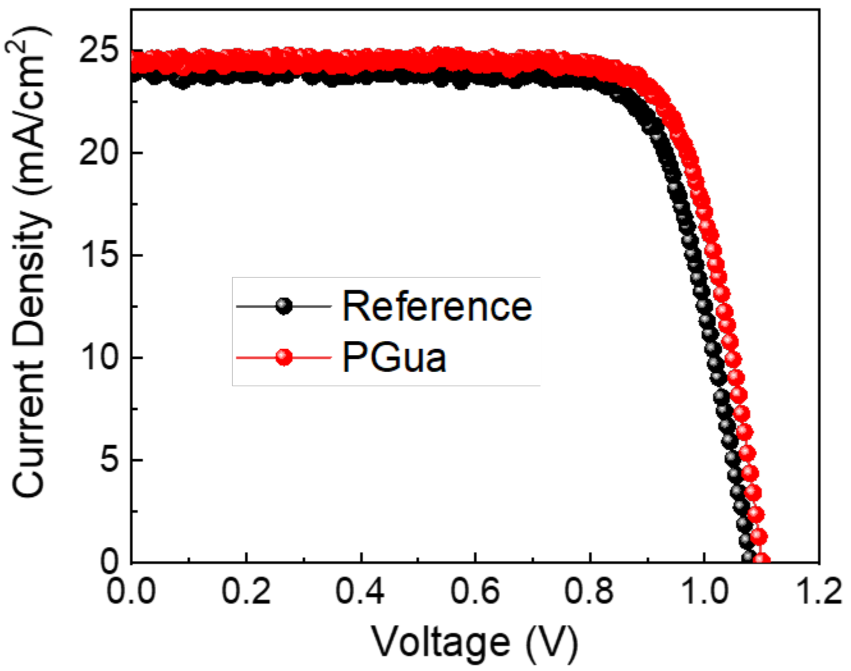

Figure S18. JV-curves of the champion $MAPbI_3 \cdot 0.05PbI_2$ devices with and without PGua treatment.

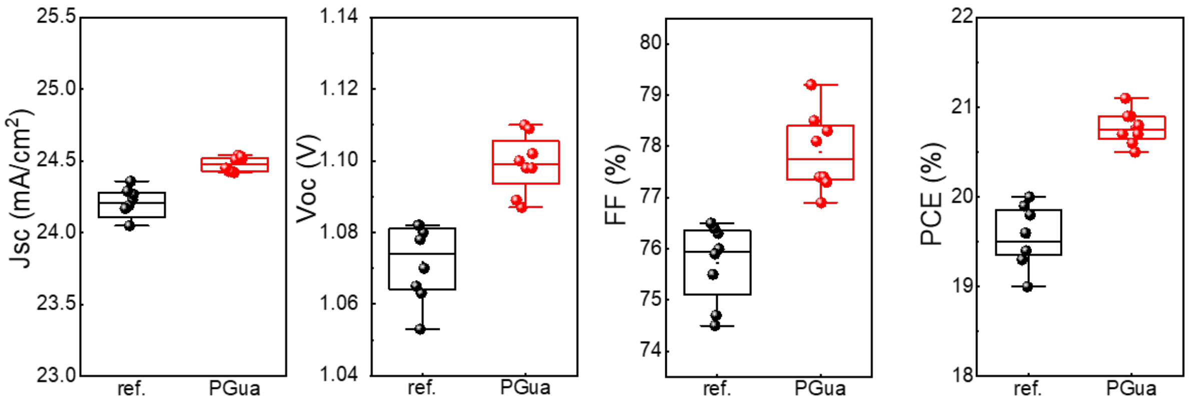

Figure S19. JV-characteristics of the $MAPbI_3 \cdot 0.05PbI_2$ PSCs with and without PGua treatment.

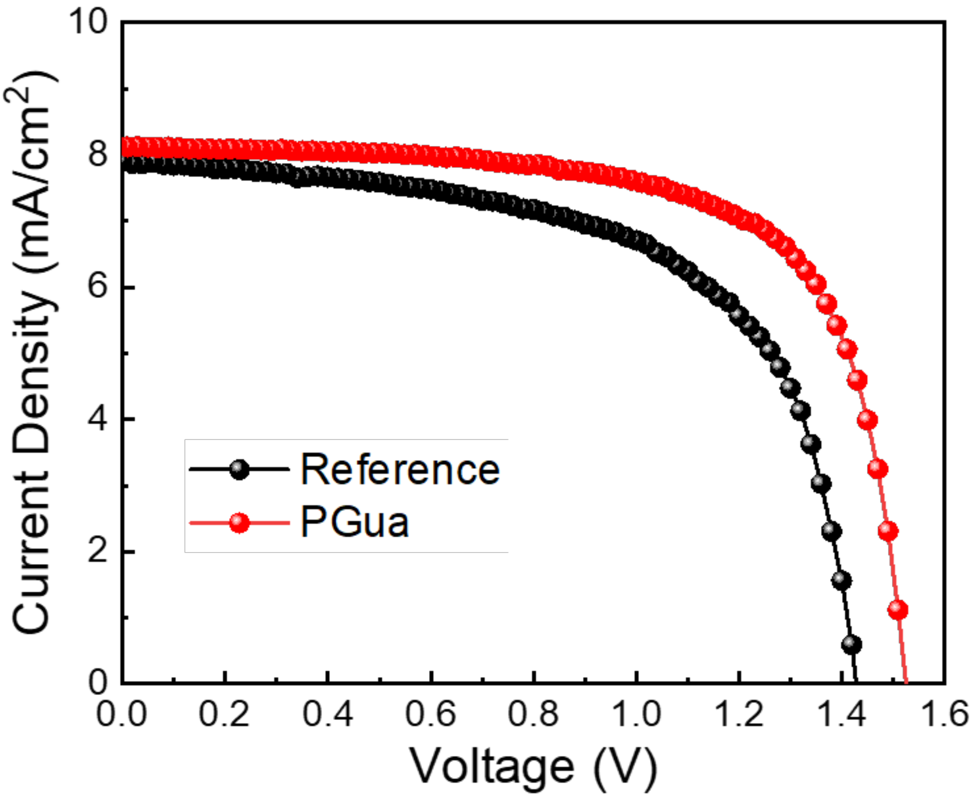

Figure S20. JV-curves of the champion $MAPbBr_3 \cdot 0.05PbBr_2$ devices with and without PGua treatment.

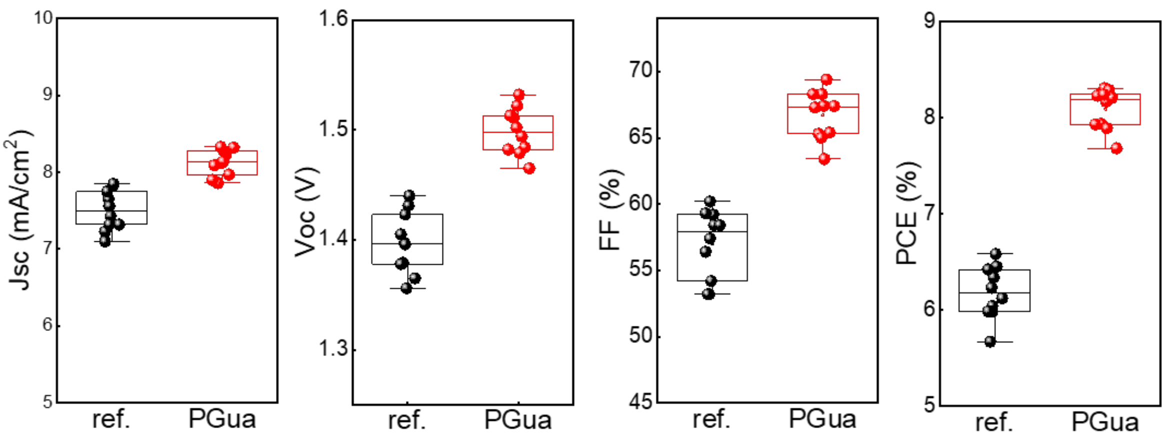

Figure S21. JV-characteristics of the $MAPbBr_3 \cdot 0.05PbBr_2$ PSCs with and without PGua treatment.

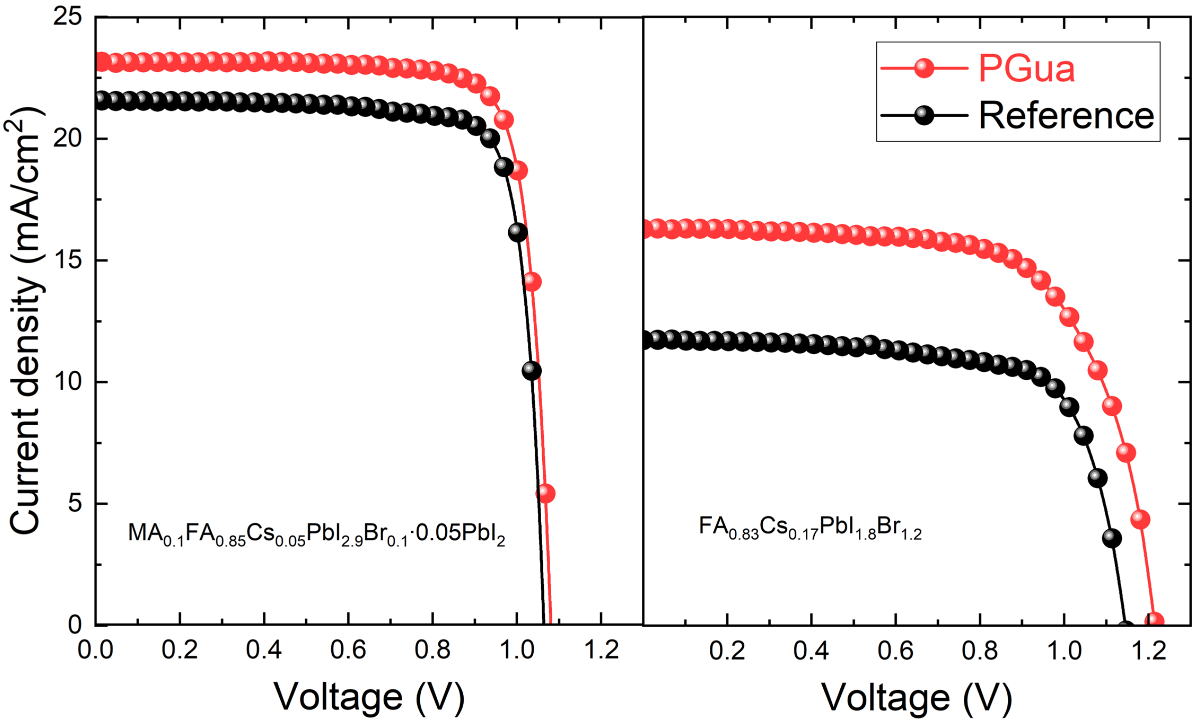

Figure S22. JV-curves of the champion p-i-n devices made with (left) triple-cation and (right) double-cation perovskites with and without addition of PGua.

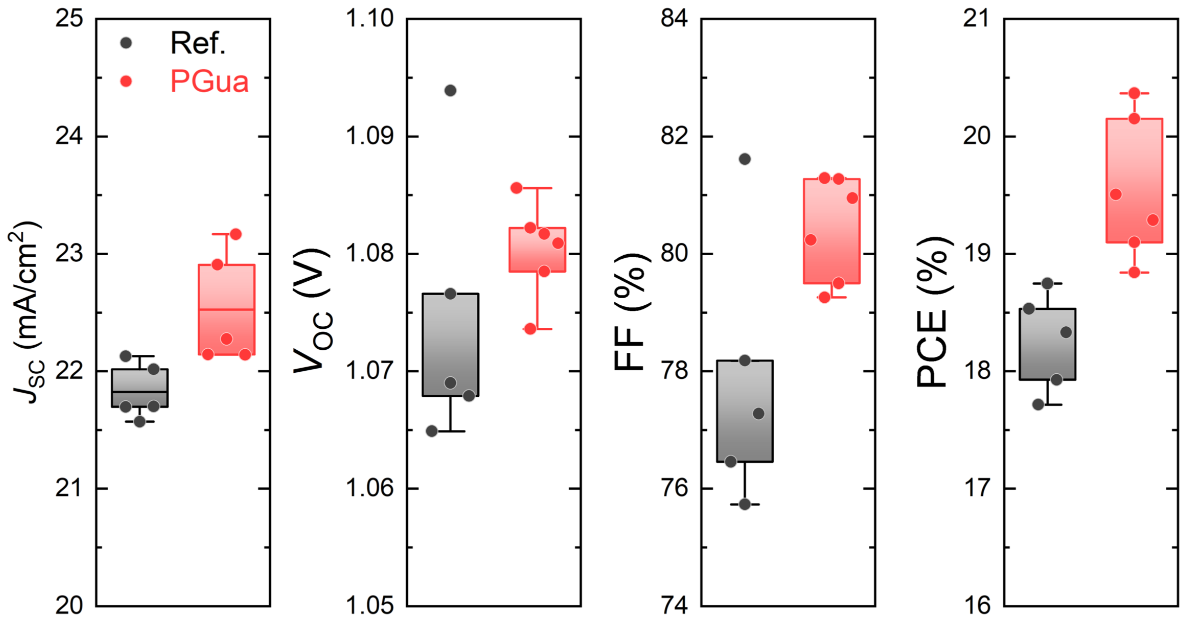

Figure S23. JV-characteristics of the manufactured p-i-n PSCs with and without an addition of PGua having $MA_{0.1}FA_{0.85}Cs_{0.05}PbI_{2.9}Br_{0.1}\cdot 0.05PbI_2$ perovskite.

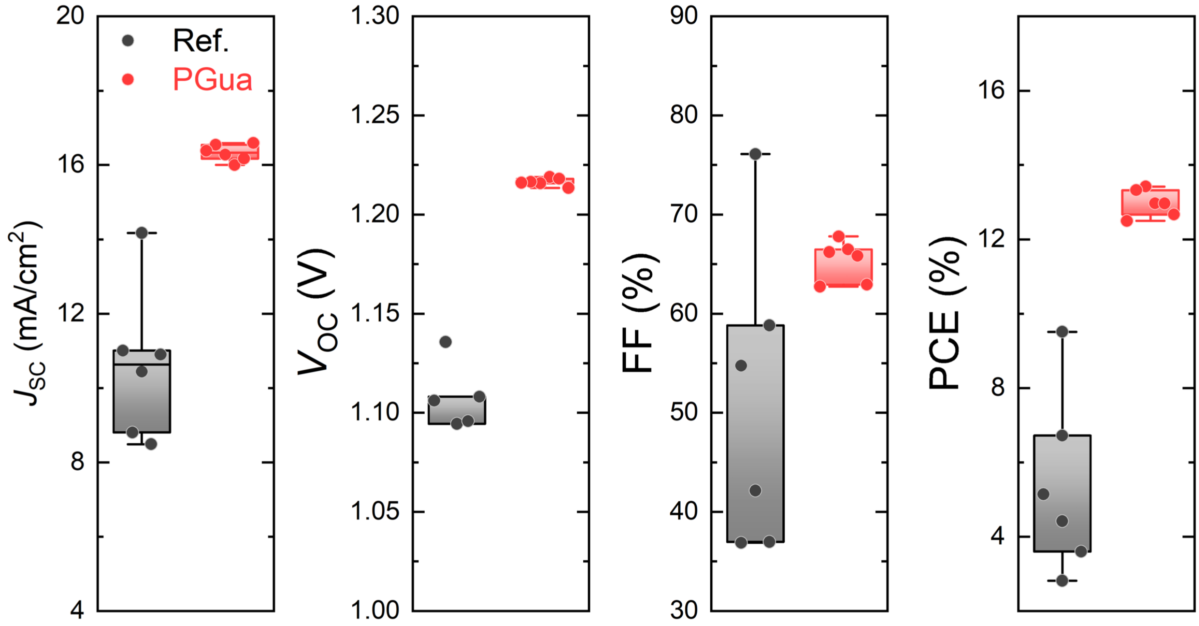

Figure S24. JV-characteristics of the manufactured p-i-n PSCs with and without an addition of PGua having $FA_{0.83}Cs_{0.17}PbI_{1.8}Br_{1.2}$ perovskite.

Reference

1. Staub, F.; Hempel, H., Hebig, J. C.; Mock, J.; Paetzold, U. W.; Rau, U.; Unold, T.; Kirchartz, T. *Phys. Rev. Applied* **2016**, *6*, 044017.
2. Perdew, J. P.; Burke, K. Ernzerhof. M. *Phys. Rev. Lett.* **1996**, *77*, 3865-3868.
3. Poglitsh, A.; Weber, D. *J. Chem. Phys.* **1987**, *87*, 6373-6378.
4. Meggiolaro, D.; Mosconi, E.; Proppe, A. H. Quintero-Bermudez, R. Kelley, S. O.; Sargent, E. H.; De Angelis, F. *ACS Energy Lett.* **2019**, *4*, 2181-2184.
45 Giannozzi, P.; Baroni, S.; Bonini, N.; Calandra, M.; Car, R.; Cavazzoni, C.; Ceresoli, D.; Chiarotti, G. L.; Cococcioni, M.; DabO, I.; *J. Phys.: Condens. Matter* **2009**, *21*, 395502.
6. Smith, D. G. A.; Burns, L. A.; Patkowski, K.; Sherrill, C. D. *J. Phys. Chem. Lett.* **2016**, *7*, 2197-2203.
7. Gražulis, S.; Chataigner, D.; Downs. R. T.; Yokochi. A. F. T.; Quiró, M.; Lutterotti, L.; Manakova, L. Manakova, E.; Butkus, J.; Moeck, P.; Le Bail, A. *J. Appl. Cryst.* **2009**, *42*, 726-729
8. Frisch, M. J. et al. Gaussian 09, Revision A.02, Gaussian, Inc., Wallingford CT, **2016**.
9. Becke, A. D. *J. Chem. Phys.* **1993**, *98*, 5648−5652.
10. Grimme, S.; Antiny, J.; Ehrlich, S.; Krieg, H. *J. Chem. Phys.* **2010**, *132*, 154104.
